# Colorectal cancer cells respond differentially to autophagy inhibition *in vivo*

**DOI:** 10.1038/s41598-019-47659-7

**Published:** 2019-08-05

**Authors:** Annie Lauzier, Josiann Normandeau-Guimond, Vanessa Vaillancourt-Lavigueur, Vincent Boivin, Martine Charbonneau, Nathalie Rivard, Michelle S. Scott, Claire M. Dubois, Steve Jean

**Affiliations:** 1 Faculté de Médecine et des Sciences de la Santé, Université de Sherbrooke, 3201, Rue Jean Mignault, Sherbrooke, Québec J1E 4K8 Canada; 2 Department of Anatomy and Cell Biology, Université de Sherbrooke, 3201, Rue Jean Mignault, Sherbrooke, Québec J1E 4K8 Canada; 3 Department of Biochemistry, Université de Sherbrooke, 3201, Rue Jean Mignault, Sherbrooke, Québec J1E 4K8 Canada

**Keywords:** Colon cancer, Macroautophagy

## Abstract

Autophagy has both tumor-promoting and -suppressing effects in cancer, including colorectal cancer (CRC), with transformed cells often exhibiting high autophagic flux. In established tumors, autophagy inhibition can lead to opposite responses resulting in either tumor cell death or hyperproliferation. The functional mechanisms underlying these differences are poorly understood. The present study aimed to investigate the relationship between the autophagic capacities of CRC cells and their sensitivities to autophagy inhibition. All studied CRC cell lines showed high basal autophagic flux. However, only HCT116 and Caco-2/15 cells displayed regulated autophagic flux upon starvation. Knockdown of ATG5 (which disrupts autophagosome elongation) or RAB21 (which decreases autophagosome/lysosome fusion) had little effect on CRC cell proliferation *in vitro*. Nonetheless, inhibition of autophagy *in vivo* had a substantial cell line-dependent impact on tumor growth, with some cells displaying decreased (HCT116 and Caco-2/15) or increased (SW480 and LoVo) proliferation. RNA sequencing and Western blot analyses in hyperproliferative SW480 tumors revealed that the mTORC2 and AKT pathways were hyperactivated following autophagy impairment. Inhibition of either mTOR or AKT activities rescued the observed hyperproliferation in autophagy-inhibited SW480 and reduced tumor growth. These results highlight that autophagy inhibition can lead, in specific cellular contexts, to compensatory mechanisms promoting tumor growth.

## Introduction

Macro-autophagy (hereafter referred to as autophagy) is defined as the lysosomal degradation and recycling of cytosolic constituents^1^. Autophagy is a multistep process that requires the generation of a double-membrane organelle called the autophagosome^2^. Cytosolic proteins and organelles are targeted inside autophagosomes during their expansion, and once completed, the latter mature to fuse with endolysosomes, where they are degraded and their content recycled^2,3^.

Autophagy is an essential homeostatic and stress response mechanism that is conserved across all eukaryotes^4^. As such, it protects cells from nutrient deprivation or oxidative, genotoxic and proteotoxic stresses, amongst others^5^. In accordance with these roles in cell homeostasis and adaptation, autophagic dysfunctions are associated with a wide range of human diseases^6^. In normal cells, autophagy protects against transformation^5,7^. Tumor suppressing capabilities of autophagy are exemplified by the fact that deletions of numerous core autophagy genes lead to increased tumor formation in a variety of tissues in mouse models^5,8–10^. Moreover, in humans, the essential autophagy gene *ATG6* is a haplo-insufficient tumor suppressor gene^11^. Autophagy is believed to protect cells from transformation by i) guarding against stress, ii) promoting anti-cancer immunity, iii) enhancing DNA damage responses, iii) minimizing aneuploidy and inflammation and iv) promoting oncogene-induced senescence^5^. Although autophagy is often upregulated in established tumors, its importance in tumor development or as a therapeutic target nevertheless remains more contentious, and is highly context-dependent, depending on the type of cancer and their genetic backgrounds, among other factors^12^.

Early studies investigating the role of autophagy in cancer focused predominantly on Ras-driven cancer models^13–15^. Using these models, it was initially proposed that Ras-driven cancers were addicted to autophagy both *in vitro* and *in vivo*^14,16^. Recent studies have challenged this model, and it is now acknowledged that autophagy addiction of Ras-driven cancers is restricted to a small subset of cancers^17–19^. Nevertheless, multiple mouse studies have demonstrated that autophagy inhibition results in decreased cancer cell proliferation and enhanced mouse survival^20–23^. At the cellular level, autophagy inhibition leads to metabolic changes culminating in decreased oxidative phosphorylation and improper lipid homeostasis and, in other settings, is associated with governing the balance between apoptosis and necroptosis^24,25^. The genetic background of tumors has also been found to play a key role in tumor responses. As such, in PDAC models, complete *TP53* loss was found to promote proliferation of autophagy-deficient cells^26^, while loss of *TP53* heterozygosity led to an opposite effect, with decreased tumor proliferation^27^. This context-dependent impact of autophagy inhibition in cancer was further exemplified by studies performed in *Drosophila*, in which diverse oncogenic drivers were shown to be differentially impacted by autophagy inhibition^28^. Finally, non-cell autonomous autophagy in cancer-neighboring cells greatly influence cancer progression^29,30^. These examples highlight the context-dependency of autophagy inhibition in cancer, and the necessity to better define the molecular determinants leading to these differential responses.

In colorectal cancer (CRC), the impact of autophagy inhibition has only recently been studied^31^. Various CRC cell lines have shown diverse sensitivities to pharmaceutical autophagy inhibition^32–36^, although through CRISPR/Cas9 genome wide screens, most CRC cell lines were shown to be insensitive to genetic ablation of autophagy in normal culture conditions^37^. In contrast, *in vivo* evidence suggests a role for autophagy during CRC development, with autophagy being active at early stages of CRC formation^20^. In the classical *Apc*^Min/+^ mouse model, autophagy inhibition was found to lead to intestinal dysbiosis, which prevented tumor growth through a CD8-mediated immune response^20^. Altogether, these *in vivo* evidences suggest that autophagy inhibition in CRC could be beneficial in patients.

Given the potential benefits of targeting autophagy in CRC patients, we revisited the link between autophagic functions in CRC cells and their response to autophagy inhibition both *in vitro* and *in vivo*, with the goal of identifying functional or genetic determinants of cell responses to autophagy inhibition. Results herein reveal that autophagy inhibition did not affect viability of CRC cells under nutrient-rich conditions in culture. However, cell line-specific responses to autophagy inhibition were identified *in vivo*, which correlated with the ability of cell lines to upregulate or not their autophagic flux. Importantly, mTOR reactivation was identified as an important cellular event leading to hyperproliferation of autophagy-deficient SW480 cells. These findings further highlight the context-dependent effects of autophagy inhibition in cancer cells and the importance of gaining a better understanding of the molecular processes impacted by autophagy inhibition in cancer cells.

## Results

### Colorectal cancer cells display different autophagic flux

While multiple studies have shown that transformed cells feature high autophagic flux^15,16^, the correlation between autophagic flux and cell sensitivity to autophagy inhibition is poorly described, with very few studies having comprehensively compared autophagic flux between human CRC cell lines. Hence, the autophagic flux of various CRC cells with different genetic alterations and *TP53* status were compared. To select the various CRC cell lines to test, emphasis was put on driver mutations, (KRAS, BRAF or PI3KCA mutations), p53 status (WT or mutated) and MSI status. Seven cell lines with various and independent alterations in these genes were hence selected (Supplemental Table 1). Autophagic flux was assessed by several complementing approaches^38–40^ in order to 1) carefully establish basal autophagy levels and 2) measure the ability of CRC cells to further induce autophagy upon stress.

**Figure 1:**
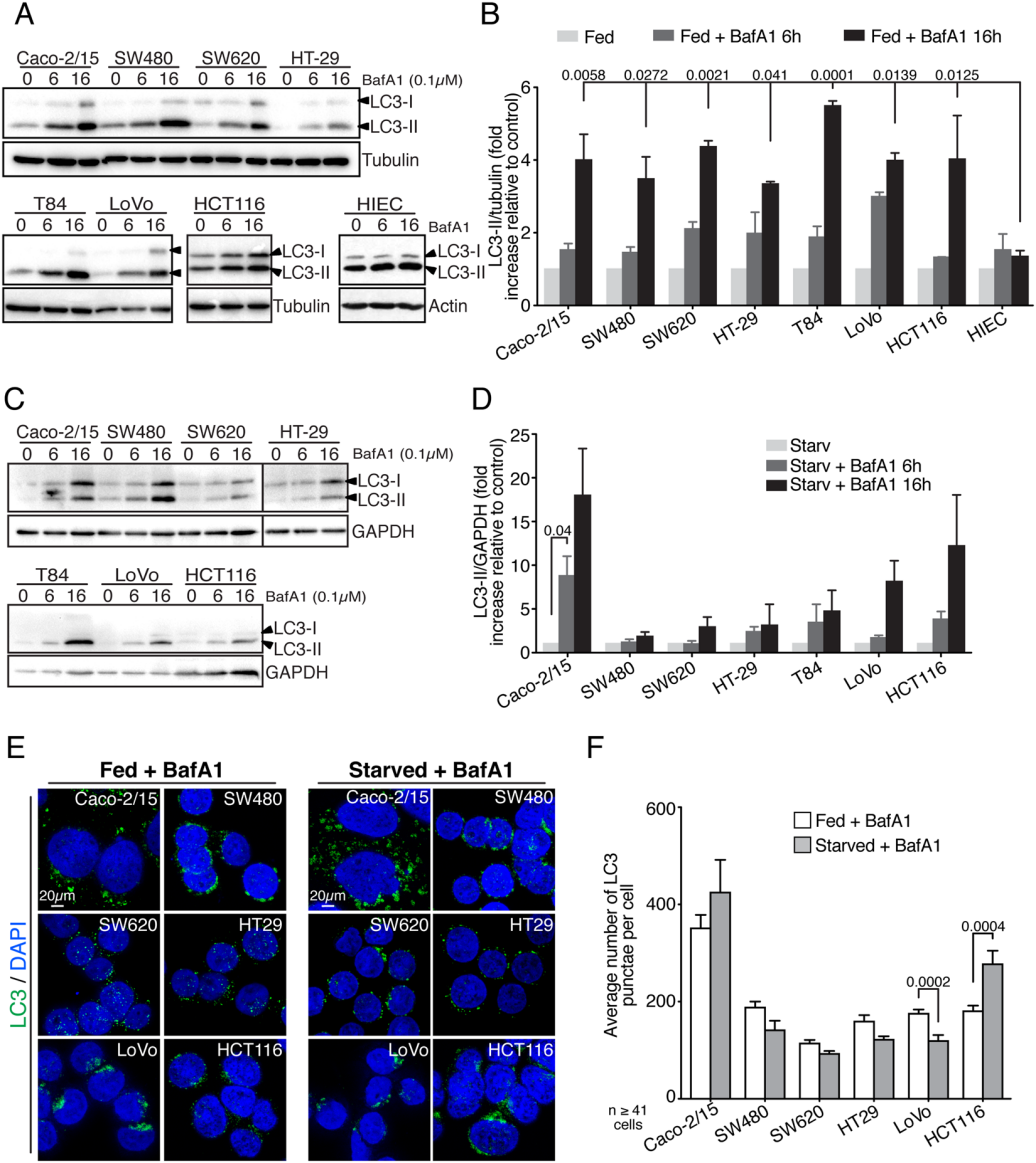
Colorectal cancer cells differentially modulate autophagy upon stress. Autophagic functions were measured by anti-LC3 immunoblot analysis of CRC cells treated with Bafilomycin A1 in (**A**) full media or (**C**) glucose starvation media. Western blot quantifications of at least three independent experiments in (**B**) full media or (**D**) under glucose starvation; mean ± SEM. As can be seen, Caco-2/15 and HCT116 both upregulated autophagosome synthesis upon glucose starvation. (**E**) Immunofluorescence analysis of endogenous LC3 under fed or glucose starvation with BafilomycinA1 treatment. (**F**) Per cell quantification of the average number of LC3 punctae (n = at least 41 cells in 3 independent experiments); mean ± SEM. Statistical analysis was performed using (**B**,**D** and **F**) One-way Anova followed by Holm Sidak multiple comparisons test. Images from different gels were separated by spaces (**A**) or a line (**C**). Full-length blots are presented in Supplementary Fig. 6.

LC3 lipidation was first monitored as a general means to assess autophagy. However, since steady-state LC3-II levels do not correlate with autophagic activity^41^, autophagic flux was blocked with Bafilomycin A1 (a known inhibitor of lysosomal functions) and the accumulation of LC3-II monitored by immunoblotting in both complete nutrient (fed) or glucose-starved conditions and quantified from multiple independent repeats. Although cell lines exhibited different steady-state LC3-II levels under standard growth conditions (Fig. S1A and^42^), all accumulated LC3-II at similar rates, with no statistical differences detected between cell lines after a 16 hours BafA1 treatment (Fig. 1A,B). Importantly, undifferentiated normal human intestinal epithelial cells (HIEC)^42^ did not accumulate considerable amounts of LC3 upon BafA1 treatment, compared to CRC cell lines (Fig. 1A,B), indicating that CRC cell lines have higher autophagic flux then normal epithelial cells. These experiments were next repeated under glucose starvation (full starvation being toxic) and, surprisingly, Caco-2/15 and HCT116 cells showed the largest accumulation of LC3-II after 16 hours of Bafilomycin A1 treatment (Fig. 1C,D). As such, Caco-2/15 and HCT116 showed respectively a 17 fold and a 13 fold increase under glucose starvation versus a 3 and a 4 fold increase in full nutrient condition. All other cell lines did not show such striking differences between starved and normal growth conditions. HIEC cells were not monitored given their high sensitivity to glucose starvation. To corroborate the immunoblot analysis, we performed immunofluorescence experiments on endogenous LC3^38,40^. Notably, only Caco-2/15 and HCT116 cells displayed the ability to induce their autophagic flux upon starvation compared to the other cell lines (Figs 1E,F and S1B and C), thus supporting the Western blot data.

**Figure 2:**
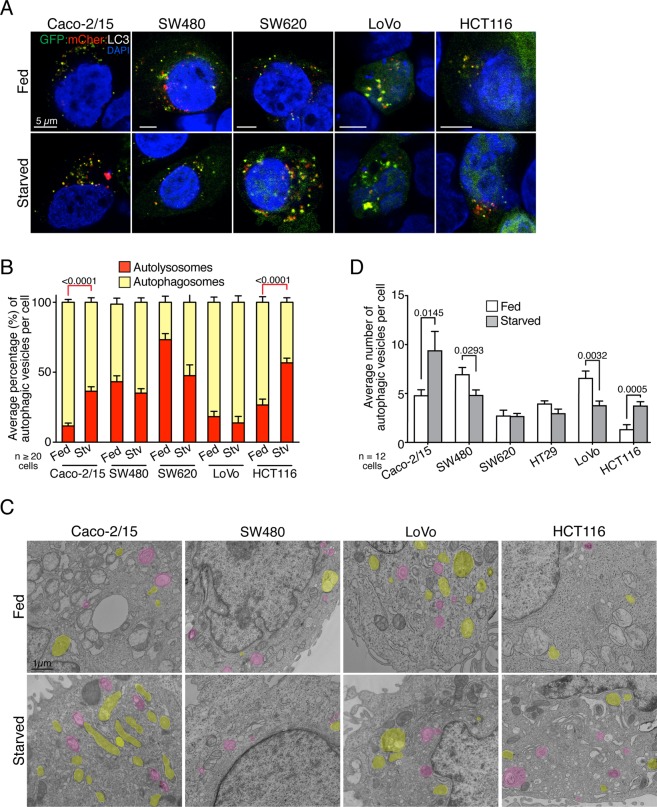
Colorectal cancer cells feature different autophagic flux. mCherry:GFP:Atg8 analysis of autophagic flux in the various CRC cell lines. (**A**) Representative images of mCherry:GFP:Atg8 transfected cells under full media or glucose starvation. (**B**) Per cell average percentage of autophagosomes and autolysosomes (n = 20 to 65 cells per condition from 3 independent experiments); mean ± SEM. (**C**) Representative images of TEM analysis in full media or under glucose starvation. Pink highlighted objects represent autophagosomes while yellow highlighted objects are lysosomes/autolysosomes. (**D**) Number of autophagic vesicles per cell averaged from twelve cells. Only double-membrane vesicles were counted as autophagosomes and electron dense vesicular structures were counted as autolysosomes; mean ± SEM. Statistical analysis was performed using (**B**) Two-way ANOVA followed by Tukey’s multiple comparison test or (**D**) One-way ANOVA followed by Kruskal-Wallis multiple comparisons test.

To more directly compare autophagic flux in CRC cell lines, GFP:mCherry:LC3 flux reporter studies were performed under fed and glucose starvation conditions. This technique allows discerning between autophagosomes (yellow) and autolysosomes (red) due to the loss of GFP fluorescence at acidic pH. Hence, an increase in the percentage of autolysosomes (red) represents increased flux^38,39^. HT29 and T84 cells were not analyzed given the poor localization of the probe in these cells. This approach highlighted varying ratios of autolysosomes/autophagosomes in the different cell lines. However, it demonstrated an increased flux in starved Caco-2/15 and HCT116 cells, illustrated by the significant increase in red labelled autolysosomes (Fig. 2A,B). Lastly, transmission electron microscopy (TEM) was performed to unequivocally assess the types of autophagic vesicles present in CRC cell lines and to further strengthen the above findings that starved Caco-2/15 and HCT116 cells upregulated their flux. TEM analysis demonstrated that CRC cells exhibited similar numbers of autophagosomes under steady state conditions, although HCT116 cells displayed a smaller number of autophagic structures and autophagosomes in these conditions (Fig. 2C,D, Fig. S1C and D). Nonetheless, after 4 h of glucose starvation, Caco-2/15 and HCT116 cells showed increased autophagic structures, whereas the number of autophagic structures in SW480 and LoVo cells was decreased (Fig. 2C,D, Fig. S1D and E). Collectively, these results show that CRC cell lines harbor comparable and elevated autophagic activities in normal conditions. The data also indicate that two cell lines can differentially adapt their autophagic response to nutrient stress, a characteristic not previously described for CRC cell lines.

### Colorectal cancer cells are insensitive to autophagy inhibition *in vitro*

Multiple studies have assessed CRC cell sensitivity to autophagy inhibition^32–36^ with most findings concluding that autophagy was required for CRC cell growth. Unfortunately, the majority of these studies relied on the use of chloroquine to reach this conclusion. Given that the effects of chloroquine on cancer cells were recently demonstrated to be independent of its effect on autophagy^17,43^, autophagy requirements were therefore revisited herein in various CRC cell lines using genetic approaches.

Two approaches of autophagy inhibition were compared, namely blocking autophagosome formation versus blocking autophagosome-lysosome fusion, by respectively targeting *ATG5* and *RAB21* expression through RNA interference, using previously validated siRNA^44,45^. RAB21 was selected given its ability to mediate the sorting of VAMP8 to endolysosomes^44^, the latter being required for autophagosome-lysosome fusion^46^. We first wanted to validate the established role of *RAB21*^44^ in the regulation of autophagy in CRC cell lines. Knockdown of *RAB21* by small interfering RNA resulted in LC3-II accumulation in most CRC cell lines (Fig. S2A). LC3-II accumulation was masked by Bafilomycin A1 treatment (Fig. S2A). These results are similar with our previous report^44^, and demonstrate the necessity of *RAB21* for normal autophagy in CRC cells. Of note, the impact of *RAB21* loss of function was similar to *VAMP8* depletion, further demonstrating a requirement for *RAB21* in autophagosome-lysosome fusion in most CRC cells^44^. However, knockdown of *RAB21* had no effect in T84 cells. This could hypothetically be caused by an increased reliance on YKT6/STX7 functions in these cells^47–49^. Thus, T84 cells were not further studied.

We next tested the impact of *ATG5* or *RAB21* depletion on CRC cell viability in normal growth conditions, through a resazurin-based assay. Silencing of *RAB21* or *ATG5* (Fig. S2B) had no effect on CRC cell viability in normal growth conditions (Fig. S3A) in accordance with other recent studies^17,37^, albeit contrary to others^34^. Since CRC cells undergoing various stresses may potentially require autophagy to alleviate these stresses, oxaliplatin and nutrient starvation were therefore used to induce a genotoxic or a nutrient stress, respectively, in CRC cells. Although glucose starvation reduced growth by over 50% in all cell lines (Fig. S3B), the concomitant inhibition of autophagy had minimal additive effect on cell growth (Fig. S3C and D). Similarly, autophagy impairment did not affect cell growth in oxaliplatin-treated CRC cells (Fig. S3B to D), as also demonstrated recently^17^. The effect of autophagy inhibition on the proliferative capacity of CRC cells following stress was further explored using clonogenic survival assays. Again, *ATG5* or *RAB21* depletion did not reduce the capacity of CRC cell lines to proliferate either alone or in combination with withdrawal of growth factors (Fig. S3E). Glucose starvation was not performed, since it affected cell viability too strongly. Altogether, the above data indicate that CRC cells do not require *ATG5* and *RAB21* for survival *in vitro*.

### *ATG5* and *RAB21* depletion in CRC cells differentially affects tumor growth

**Figure 3:**
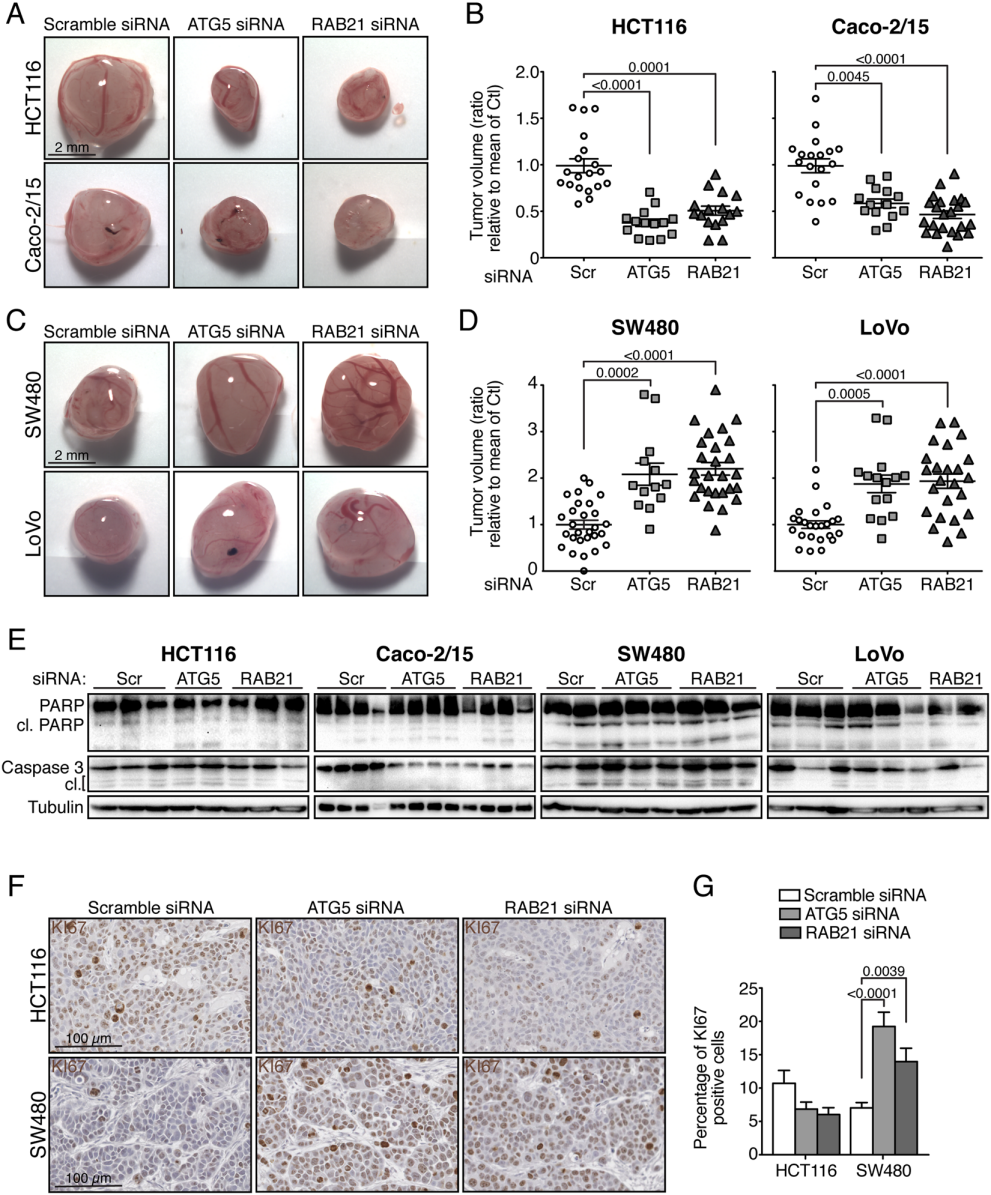
Autophagy differentially affects tumor growth *in vivo*. Tumor formation of various autophagy-inhibited CRC cell lines in chicken chorioallantoic membranes (CAM). Images of (**A**) HCT116 and Caco-2/15 or (**C**) SW480 and LoVo, treated with scramble, ATG5 and RAB21 siRNA implanted in CAMs and grown for 7 days post-implantation. (**B**) Quantification of tumor growth depicted in A (n = at least 13 tumors from 3 independent experiments); mean ± SEM. (**D**) Quantification of tumor growth depicted in C (n = at least 14 tumors from 3 independent experiments); mean ± SEM. Apoptosis was not strongly induced in ATG5 and RAB21 knockdown CRC cells grown in CAMs. (**E**) Immunoblot analysis of cleaved PARP and cleaved Caspase 3 in individual tumors generated with various CRC cell lines depleted for ATG5 and RAB21. Proliferation was increased in autophagy-inhibited SW480 cell tumors generated in CAMs. (**F**) Representative immunohistochemistry images of the proliferation marker KI67 in HCT116 and SW480 tumors extracted 5 days post-implantation. Slides were counterstained with hematoxylin/eosin. (**G**) Quantification of KI67-positive cells from four random fields in 2 to 5 tumors per condition; mean ± SEM. Statistical analysis (**B**,**D** and **G**) was performed using One-way ANOVA followed by Kruskal-Wallis multiple comparisons test. Full-length blots are presented in Supplementary Fig. 6.

Implantation of cells in chicken chorioallantoic membrane (CAM) have successfully been used as an *in vivo* system to study tumorigenesis in a variety of cancer models, including CRC^50^. We therefore tested the capacity of autophagy-inhibited CRC cell lines to form tumors after grafting on CAM. siRNA-transfected CRC cells were implanted 48 hours following transfection, in which gene knockdown was validated for all experiments (Fig. S2B). All CRC cell lines transfected with control siRNA were able to develop tumor masses, visible at 3 days post-grafting, when deposited on the CAM (Fig. 3A to D and S4A to C). These tumor masses were fairly similar in size within each cell line, although were histologically different between cell lines (Fig. S5A). Contrary to results obtained *in vitro*, depletion of either *ATG5* or *RAB21* markedly affected tumor growth (Fig. 3A to D). HCT116 and Caco-2/15 cells, both of which exhibited high autophagic flux under glucose starvation *in vitro*, were sensitive to autophagy inhibition and showed a 50% decreased tumor growth upon *ATG5* or *RAB21* depletion (Fig. 3A,B). Surprisingly, knockdown of *ATG5* and *RAB21* in SW480 and LoVo resulted in increased tumor growth (Fig. 3C,D), whereas SW620 and HT29 cell lines were non-responsive to autophagy inhibition and grew to similar extents whether in control or treated conditions (Fig. S4A to C). Of note, although knockdown efficiencies were variable between cell lines (Fig. S2B) and did not persist over the full time course of tumor growth, increased p62 staining was observed in *ATG5*- and *RAB21*-depleted CRC tumors, suggesting that autophagy was impaired, in our CAM model (Fig. S4D to G). Collectively, these results indicate that *ATG5* or *RAB21* depletion in CRC cells can either inhibit or promote tumor growth.

### Increased proliferation is observed in SW480 autophagy-deficient cells

There were no major macroscopic differences between control and *ATG5*- or *RAB21*-depleted tumors for all cell types (Fig. S5A), suggesting that the varying tumor sizes could be due to the modulation of apoptosis or proliferation. The status of apoptosis was therefore verified in individual tumors by Western blot analysis. No significant changes were observed in PARP or Caspase 3 cleavage between control and *ATG5*- or *RAB21*- depleted cells (Fig. 3E), indicating that apoptosis induction was not responsible for the decreased tumor size in HCT116 cells. However, KI-67 positive cells, a marker strongly associated with proliferating cells, were significantly more abundant in SW480-depleted tumors (Fig. 3F,G). Conversely, HCT116 cells showed a trend toward lower levels of KI-67-positive cells in *ATG5*- and *RAB21*-depleted tumors (Fig. 3F,G). These results suggest that larger tumor masses were attributable to increased proliferation, as seen in SW480, while decreased tumor size, as in HCT116, could not be linked to apoptosis activation, but potentially to increased necroptosis or to metabolic reprogramming as observed in other systems^25,51,52^.

### Different pathways are modulated by *RAB21* depletion in HCT116 and SW480 cells

**Figure 4:**
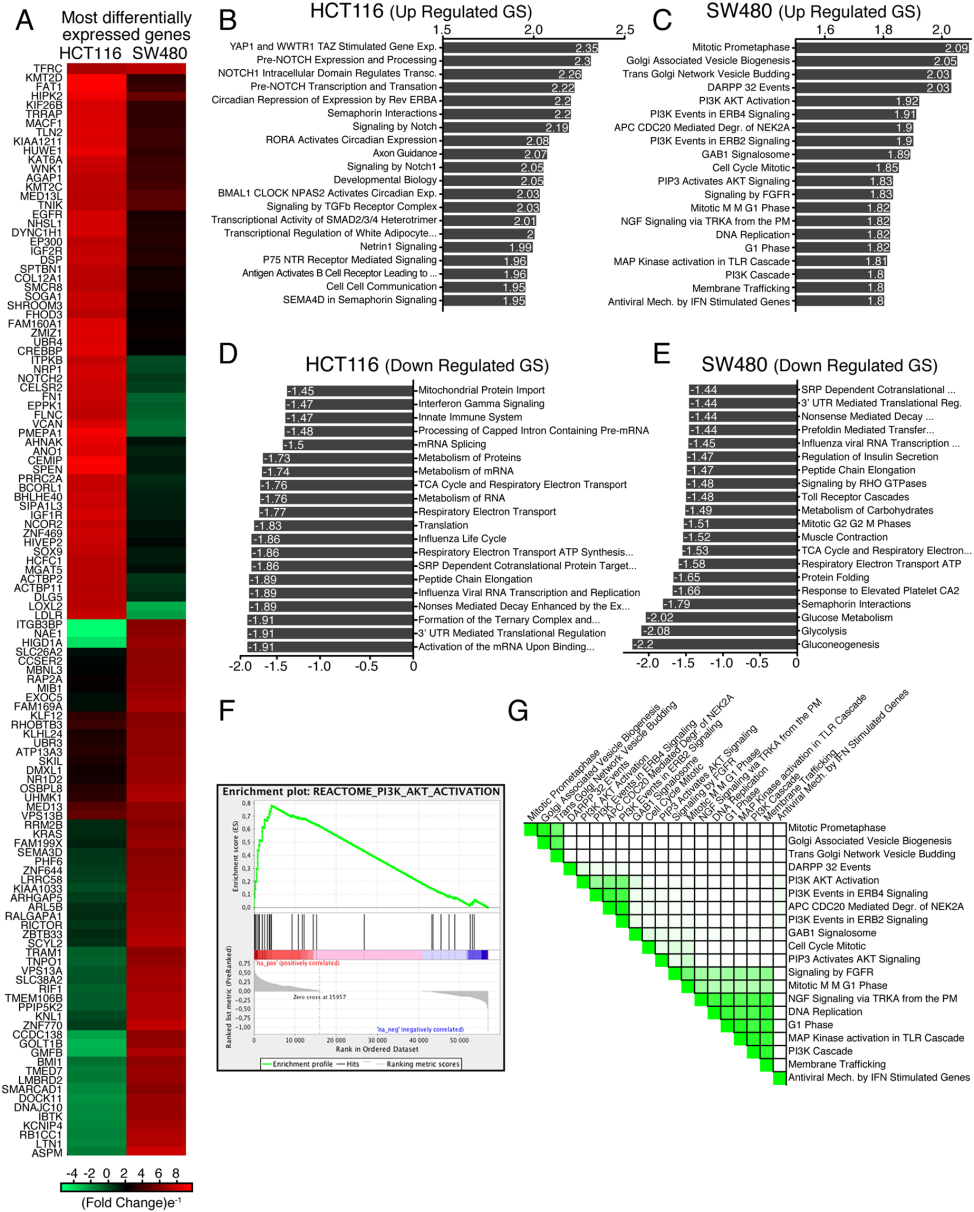
RAB21 depletion differentially affects gene expression in HCT116 and SW480 cells. (**A**) Clustered heatmap of the top 60 most differentially expressed genes in *RAB21*-depleted HCT116 and SW480 cells. (**B** to **E**) Most up/down regulated differentially expressed Reactome Gene Sets (RGS) between control and *RAB21*-depleted cells generated through a GSEA analysis and classified according to their normalized enrichment score NES score in HCT116 and SW480. All represented GSEA pathways had False Discovery Rate values inferior to 0.003. (**F**) Representative enrichment plot of *RAB21*-depleted SW480 cells. (**G**) Leading Edge analysis of the twenty most differentially upregulated pathways in *RAB21*-depleted SW480 cells.

Given the contrasting effects of autophagy inhibition on tumor growth in HCT116 and SW480 cells, and given the fact that both are driven by oncogenic KRAS mutations, we next investigated the global cellular processes modulating this opposite cellular response. A large-scale transcriptome analysis (RNA-Seq) of tumors formed in CAM from *RAB21*-depleted HCT116 and SW480 cells (Suppl. Table 2) revealed that *RAB21* knockdown resulted in highly different gene expression profiles compared to scramble control cells, this in both cell lines (Fig. 4A, only top 60 differentially expressed genes are displayed). A functional enrichment analysis of the differentially expressed genes was next performed using GSEA against the Reactome database. In HCT116, multiple NOTCH-mediated processes were upregulated in *RAB21*-depleted cells (Fig. 4B), which could explain the observed decrease in cell proliferation given the role of Notch in intestinal epithelial cell differentiation. Moreover, a large number of gene sets related to proliferation and to mitochondrial functions were also decreased in knocked-down HCT116 (Fig. 4D). This finding is in keeping with the known metabolic reprogramming observed in cells in which *ATG5* or *ATG7* inhibition affected tumor formation^22^. No differences were observed at the level of apoptotic gene expression^52^, which was consistent with the lack of increased apoptotic protein processing (Fig. 3E). To the contrary, multiple growth promoting pathways were upregulated in *RAB21*-depleted SW480 cells (Fig. 4C,F). A leading-edge analysis of the twenty most enriched pathways in SW480 tumors highlighted a large degree of overlap between the various gene sets. This was particularly evident with PI3K, at the level of AKT and mTOR signaling cascades (Fig. 4G). These observations thus highlight strikingly different responses to *RAB21*-depletion in HCT116 and SW480 cells, these differences being consistent with the growth characteristics of the respective tumor masses.

### Autophagy inhibition in SW480 results in ectopic mTOR activation

**Figure 5:**
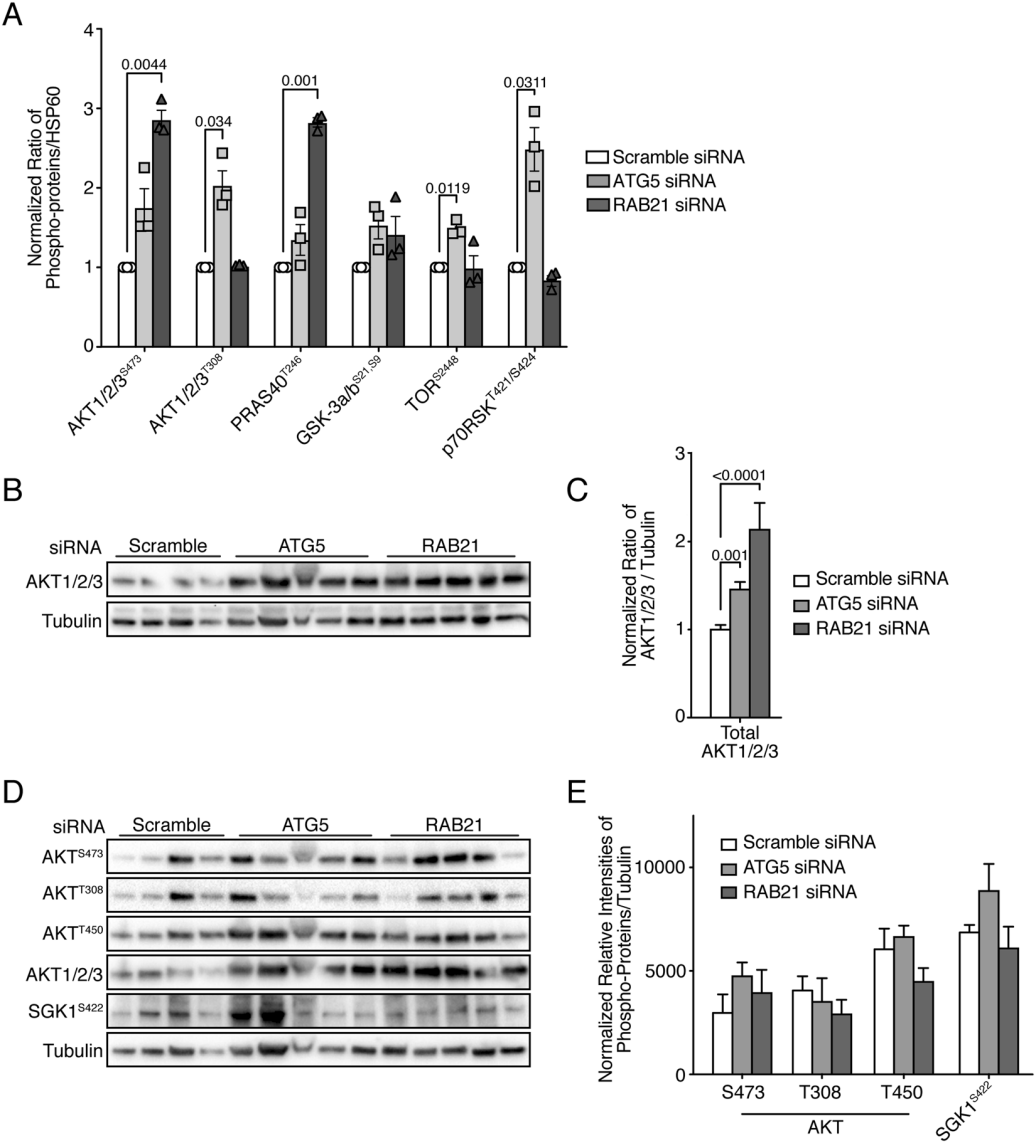
AKT expression is increased in autophagy-inhibited SW480 cells. A variety of signaling kinases were differentially phosphorylated upon autophagy inhibition. (**A**) Phosphoarray normalized ratios from three independent *ATG5*- and *RAB21*-tumors normalized to pooled scrambled control tumors. Each bar represents duplicates for each tumor. Statistical significance was determined using one sample T-test. (**B**) Immunoblot analysis of total AKT1/2/3 and tubulin from multiple SW480 tumors extracted five days post-implantation in CAMs. (**C**) Ratio of AKT1/2/3 integrated densities to Tubulin for all tumors; mean ± SEM (n ≥ 22). (**D**) Immunoblot analysis of various phosphorylation sites on AKT, SGK1^S422^ and total AKT1/2/3 and Tubulin expression from multiple independent SW480 tumors extracted five days post-implantation in CAMs. (**E**) Ratio of phosphorylated AKT or SGK1 integrated densities to Tubulin for all tumors; mean ± SEM (n ≥ 4). Statistical significance was determined using One-way ANOVA followed by Kruskal-Wallis multiple comparisons test. Full-length blots are presented in Supplementary Fig. 6.

Molecular changes occurring in cancer cells sensitive to autophagy inhibition have been thoroughly characterized in different contexts and often involve metabolic reprogramming and increased sensitivity to apoptosis or necroptosis^12,51^. However, mechanisms utilized by cells harboring increased proliferation upon autophagy inhibition remain to be defined. In order to further explore the mechanisms leading to SW480 overproliferation, the phosphorylation status of 43 kinases was examined in three independent *ATG5*- and *RAB21*-depleted SW480 tumors using the Proteome Profiler Human Phospho-Kinase Array Kit. Proliferation cues generally transit through cells via two main signaling nodes, the RAS/RAF/MEK/ERK or the PI3K/AKT signaling pathways. No shared changes in phosphorylation levels of ERK1/2, RSK1/2/3, MSK1/2, p38alpha, JNK1/2/3 or c-Jun were found between control and *ATG5* or *RAB21*-depleted tumors (Fig. S5B). On the other hand, similar trends showing increased phosphorylation of AKT1/2/3 on serine 473, as well as PRAS40 and GSK3a/b, two direct targets of the RAC-alpha serine/threonine-protein kinase (AKT), were observed in most tumors following both *ATG5* and *RAB21* knockdowns (Fig. 5A). This suggests the possible involvement of the PI3K/AKT pathway in the observed increased proliferation of SW480 following autophagy inhibition, consistent with the RNA-Seq GSEA analysis.

**Figure 6:**
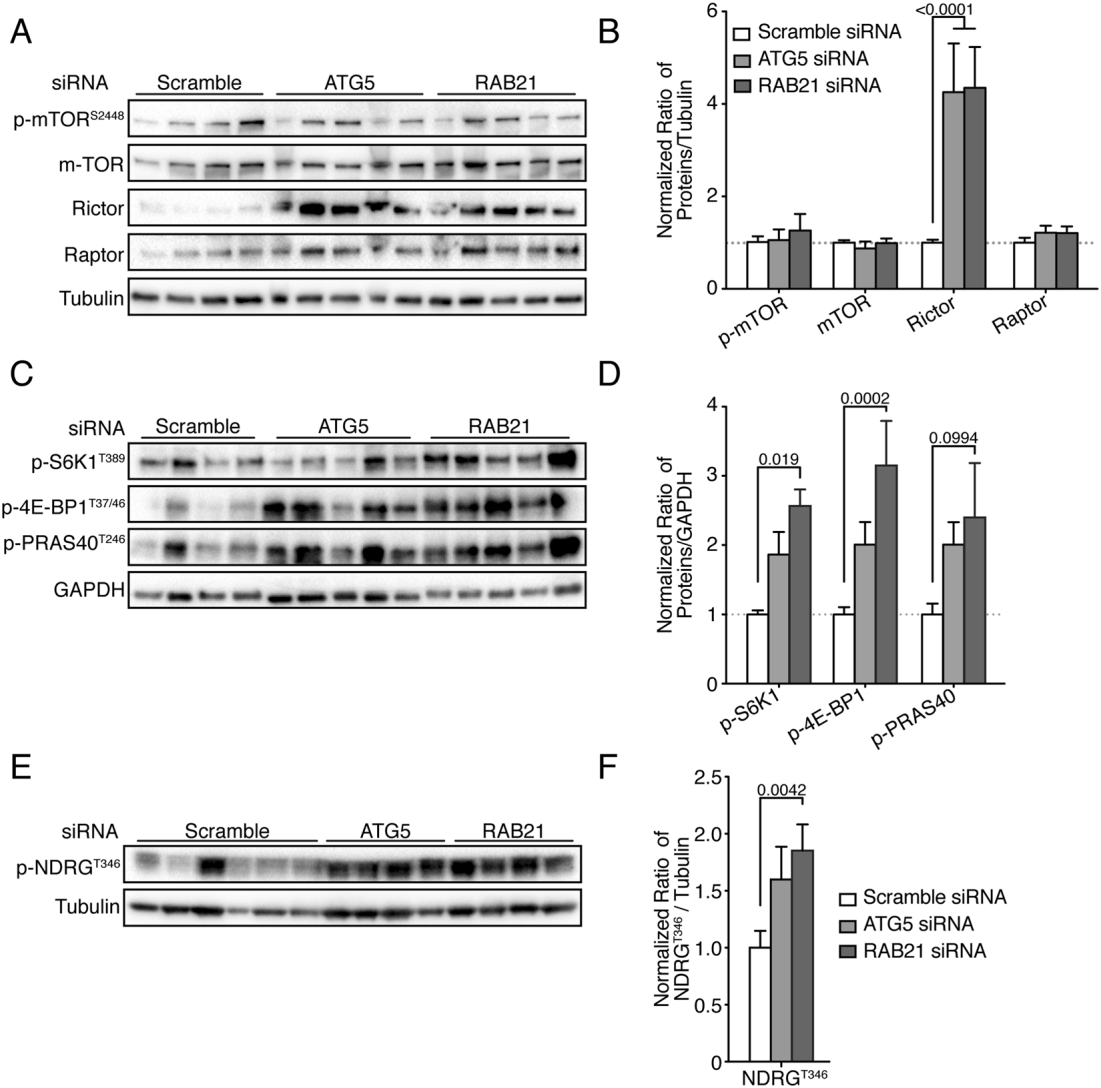
Autophagy inhibition in SW480 increases mTORC1 and mTORC2 signaling. Rictor is strongly upregulated in *ATG5* and *RAB21* knockdown SW480 tumors. (**A**) Immunoblot analysis of phosphorylated mTOR at Serine 2448 and of total mTOR, Rictor, Raptor and Tubulin from multiple independent SW480 tumors extracted five days post-implantation in CAMs. (**B**) Ratio of various integrated densities of phosphorylated and total proteins to Tubulin for at least 7 tumors per cell lines; mean ± SEM. mTORC1 activity was increased in autophagy-inefficient SW480 tumors. (**C**) Immunoblot analysis of phosphorylated S6K1^Thr389^, 4E-BP1^T37/46^, PRAS^T246^ AKT1/2/3 and GAPDH from multiple SW480 tumors extracted five days post-implantation in CAMs. (**D**) Ratio of integrated densities of phosphorylated proteins to GAPDH for all tumors; mean ± SEM (n ≥ 11). (**E**) Immunoblot analysis of phosphorylated NRDG1^T346^ and Tubulin expression from multiple SW480 tumors extracted five days post-implantation in CAMs. (**F**) Ratio of integrated densities of phosphorylated NRDG1 to Tubulin for all tumors; mean ± SEM (n ≥ 8). Statistical analysis was performed using Unpaired T Test followed by Holm Sidak multiple comparisons test. Full-length blots are presented in Supplementary Fig. 6.

An important effector of AKT on cell growth is the mTORC1 complex. A small increase in the phosphorylation of mTOR was observed, particularly in the *ATG5*-depleted tumors (Fig. 5A). Only one downstream target of mTOR, namely p70S6K, was represented in the array, and its phosphorylation status increased upon *ATG5* knockdown (Fig. 5A). To complement the above phospho-kinase array results, the expression and phosphorylation status of components of the AKT/mTOR pathway were analyzed by Western blot on proteins from a wider panel of control and siRNA-depleted SW480 tumors. In agreement with the GSEA analysis in which PI3K/AKT pathways were highly represented, a strong increase in basal pan-AKT protein levels was observed in *ATG5*- and *RAB21*-depleted tumors (Fig. 5B,C). This finding was consistent with the observed increase in AKT transcripts demonstrated by RNA-Seq (Fig. 4). The increased AKT protein level, however, did not translate into a dependable activity increase, as no increase in phosphorylation was observed on three major AKT sites in *ATG5*- and *RAB21*-depleted cells (Fig. 5D,E). Given that multiple pathways and regulatory feedback loops affect AKT activity^53^, potentially leading to a rapid phosphorylation status turnover, the analysis window on AKT may have prevented observing enhanced AKT phosphorylation. Although an increased mTOR mRNA level (Suppl. Table 2) and a slight hyperphosphorylation of the protein in the kinase array were observed, there was no consistent variation in its protein level or phosphorylation status by Western blotting in a large number of *ATG5*- and *RAB21*-depleted tumors (Fig. 6A,B). Since mTOR phosphorylation does not represent the best readout of mTOR activity^54^, well-characterized mTORC1 and mTORC2 targets were therefore monitored to more accurately assess mTORC activity. While mTORC1-associated protein RAPTOR levels did not vary, S6K1 (p70RSK) and 4E-BP1 were significantly hyper-phosphorylated upon autophagy inhibition in the vast majority of tumors (Fig. 6C,D), suggesting mTORC1 activation. PRAS40, which can be phosphorylated by both AKT and mTORC1, also showed an increased phosphorylation trend upon *ATG5* and *RAB21* knockdown (Fig. 6C,D), as observed in the phosphoprotein array results.

A marked increase in RICTOR protein levels, a component of the mTORC2 complex, was also noted in knocked down tumors. Although targets of mTORC2 complex have been less studied than those of mTORC1, they include AKT, SGK1 and the SGK1 substrate NRDG1^55^. As outlined above, AKT phosphorylation on S473 was not consistently increased in autophagy-depleted tumors, with the same trend observed for SGK1 phosphorylation (Fig. 5D,E). Again, given the multiple feedback loops regulating AKT and SGK1, phosphorylation of NRDG1 was monitored, the latter being a robust indicator of mTORC2 activity^55^. Phosphorylation of NRDG1 at T346 was increased in *ATG5* and *RAB21* knocked down tumors, indicating hyperactivated mTORC2 activity (Fig. 6E,F). Collectively, these results support the RNA-Seq findings which showed an overrepresentation of the AKT/mTOR pathway following *RAB21* depletion. Results of the kinase array and Western blot analyses hence indicate that both *ATG5* and *RAB21* depletion leads to reactivation of mTORC1 and mTORC2 signaling in SW480 cells tumors.

### AKT and mTOR inhibition rescues SW480 overproliferation-mediated by *ATG5* or *RAB21* knockdown

**Figure 7:**
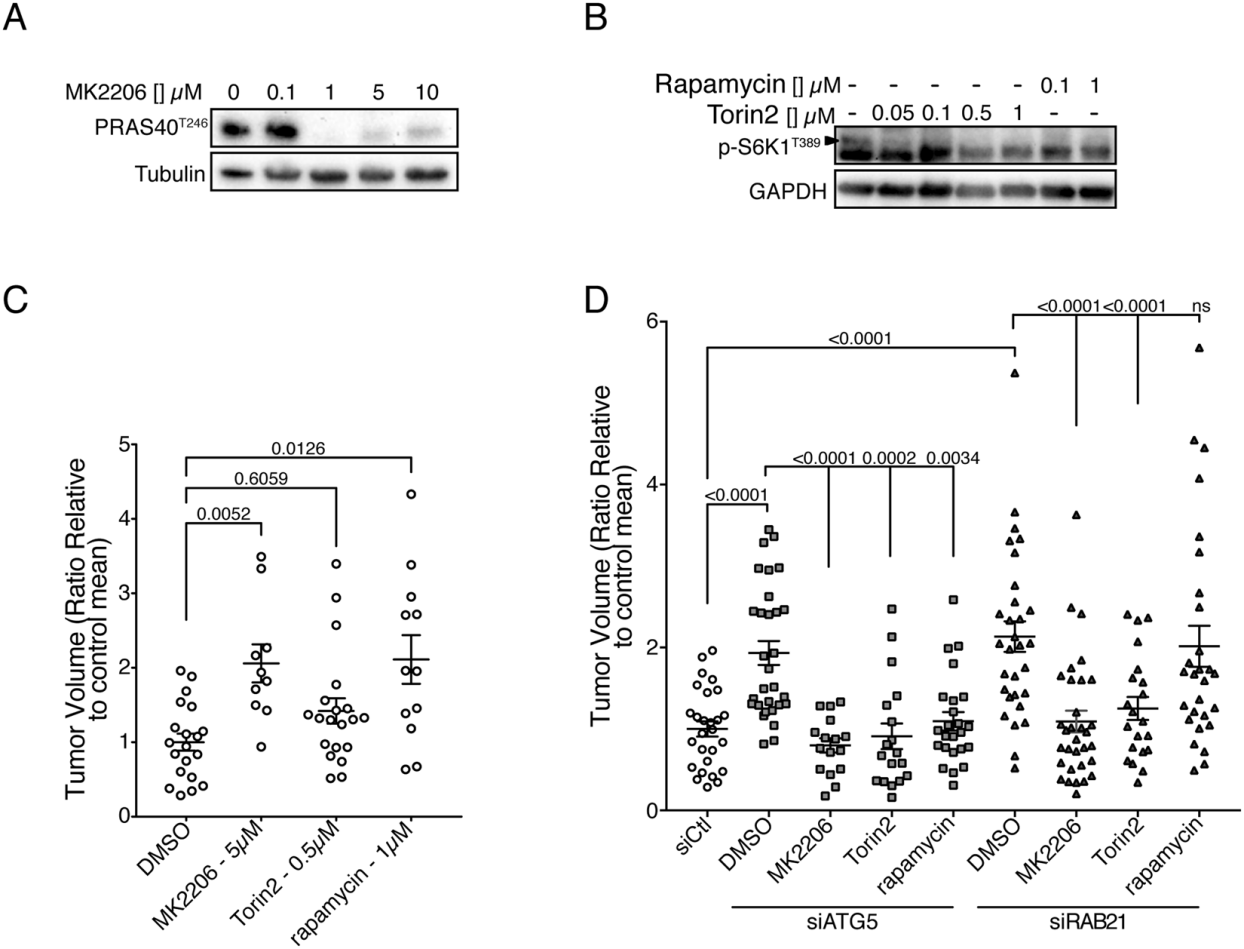
AKT and mTORC inhibition blocks SW480 hyperproliferation observed in *ATG5*- and *RAB21*-depleted cells. Efficiency validation of AKT and mTOR inhibitors. (**A**) Immunoblot analysis of phosphorylated PRAS40^T246^ upon addition of increasing concentrations of MK2206. (**B**) Immunoblot analysis of phosphorylated S6K1^T389^ upon addition of increasing concentrations of Rapamycin and Torin 2. Treatments were performed for 48 hours. (**C**) Quantification of SW480 tumor growth treated with different inhibitors. The dotplot graph depicts tumor sizes 5 days post-implantation in CAMs with drug treatments in at least 3 independent experiments; mean ± SEM. (**D**) Quantification of SW480 tumor growth treated with different inhibitors and transfected with scrambled, *ATG5* or *RAB21* siRNA. The dotplot graph depicts tumor sizes 5 days post-implantation in CAMs with drug treatments in at least 3 independent experiments; mean ± SEM. Statistical significance was determined using One-way ANOVA followed by Kruskal-Wallis multiple comparisons test. Full-length blots are presented in Supplementary Fig. 6.

Given the increased levels of AKT and the activation of the mTOR pathways in autophagy-deficient SW480 cells, we next determined whether inhibition of these pathways in conjunction with autophagy impairment could potentially slow tumor growth in our system. The allosteric AKT inhibitor MK2206 and the mTOR inhibitors Torin2 and Rapamycin, which target both mTORC1 and 2 and mTORC1 respectively, were tested. The effectiveness of these inhibitors was first confirmed in SW480 cells in culture. As expected, MK2206 reduced PRAS40 phosphorylation in a dose-dependent manner (Fig. 7A). Similarly, both Torin2 and Rapamycin prevented phosphorylation of S6K1^T389^ at all concentrations tested (Fig. 7B, upper band). The toxicity of these compounds was next tested on SW480 growth in CAM. Wild type SW480 cells were able to form tumors in the presence of the AKT and mTOR inhibitors at the selected concentrations (Fig. 7C). Surprisingly, a significant increase in tumor growth was observed upon MK2206 and Rapamycin treatments, while Torin2 treatment caused an increased trend in growth. Conversely, treatment of autophagy-deficient cells with MK2206 or Torin2 efficiently reduced tumor sizes to control levels (Fig. 7D). Rapamycin treatment also reduced tumor growth, although less effectively and was moreover ineffective in *RAB21*-depleted cells. These results demonstrate that AKT and mTORC2 strongly contribute to autophagy-deficient SW480 hyperproliferation, while mTORC1 activity does not play a major role and is not essential, at least in *RAB21*-depleted cells.

## Discussion

Autophagy inhibition was proposed as a promising approach to decrease cancer cell proliferation, especially in RAS-driven cancers^7,13^. However, recent findings have demonstrated that inhibiting autophagy can lead to tumor cell hyperproliferation^26,28^. This hyperproliferation was linked to the *TP53* status of cancer cells^26^ or to the type of oncogenic drivers^28^. In the present study, we carefully monitored autophagic flux of multiple CRC cell lines and demonstrated that these cells do not all display similar autophagic flux. Notably, HCT116 and Caco-2/15 cells were found to substantially increase their autophagic flux upon nutrient stress, compared to other CRC cells tested. Our findings, in accordance with others^17,37^, show that autophagy inhibition does not affect CRC proliferation *in vitro*. However, we show that CRC cells differentially responded to autophagy inhibition *in vivo*, with Caco-2/15 and HCT116 cells being sensitive to autophagy inhibition whereas SW480 and LoVo cells displayed increased tumor growth. Genomic and phosphoprotein analyses revealed that the hyperproliferation observed in SW480 was caused by ectopic activation of the AKT and mTOR pathways. Significantly, this hyperproliferation was mainly dependent on AKT and mTORC2 signaling, and to a lesser extent on mTORC1 signaling.

Our results are coherent with recent studies indicating that RAS-driven cancers are not all sensitive to autophagy inhibition^17,37^. As such, we did not observe growth defects *in vitro*, even under starvation or genotoxic stresses. In order to assess the *in vivo* requirements of autophagy in CRC tumor formation in multiple cell lines, we turned to chicken chorioallantoic membrane assays. Significantly, we were able to uncover differential responses to autophagy inhibition in this setting. To genetically affect autophagosome/lysosome fusion, we targeted *RAB21*^44^. RAB21 has been implicated in other cellular functions^56,57^. Hence, its effect on tumor cell growth in CAM, could be caused by its other cellular roles, and we cannot rule out this possibility. However, *RAB21* and *ATG5* shared all the same growth phenotypes and most signaling pathways analyzed were also similarly affected, indicating that *RAB21* autophagic functions must be critical for the observed effects.

All cell lines used herein were previously shown by others to be sensitive to chloroquine treatments *in vitro*^32–36^, and thus were inferred to be sensitive to autophagy inhibition. Our results show that genetic manipulation of autophagy through *ATG5* and *RAB21* RNAi does not affect CRC cell growth *in vitro*, while yielding different results *in vivo*. Since autophagy was not entirely abrogated in *ATG5*- or *RAB21*-depleted cells, the discrepancy between *in vitro* and *in vivo* responses could potentially be attributed to incomplete knockdowns, which could be insufficient to give rise to *in vitro* effects. We do not believe this to be the case, given that recent CRISPR/Cas9 genome wide screens did not identify autophagic genes to be required for viabilities of more than 500 cell lines of different origins *in vitro*^37^. Moreover, autophagy-deficient HCT116 knockouts have been generated in various contexts and, as observed in our work, did not show *in vitro* growth defects^17^. Hence, the observed *in vivo* phenotypes most likely represent higher requirements for autophagy in this setting. One caveat of the siRNA approach used here, is that knockdowns were not observed seven days post implantation. Hence, the observed growth phenotypes most probably result from early effects of *ATG5* and *RAB21* depletion on tumor growth and would presumably be stronger in the context of a prolonged knockdown (or knockout). Regardless of that limitation, autophagic flux was still reduced five days post implantation, given the increased p62 staining (Fig. S4D to G). We believe that the siRNA approach in CAMs more closely mimics autophagy inhibition in clinical settings, where autophagy would most likely not be completely abolished by drug treatments. How exactly the CAM microenvironment leads to differences in growth of autophagy-defective tumors is unknown. We believe that a combination of multiple tumor microenvironment factors is responsible for the observed changes, given that modulating nutrient or growth factors concentrations *in vitro* was not sufficient to phenocopy the CAM results.

CRC are highly heterogeneous and associated with various genomic mutations. Recent genomic efforts have allowed the classification of CRC tumors and cell lines into biologically relevant subtypes^58–62^. The cell lines used in the present study were previously assigned to various consensus molecular subtypes (CMS)^63^: CMS1 (LoVo), CMS3 (HT29) and CMS4 (HCT116, SW480, SW620, Caco-2). LoVo and HCT116 cells have the hypermutator phenotype (MSI) while the other cell lines do not. None of these associations explains the cells’ responsiveness to autophagy inhibition, nor the previously documented mutations in critical cancer-associated genes. Both cell lines in which autophagy inhibition amplified proliferation have a wild type *PIK3CA* gene, although this feature is also shared by SW620 and Caco-2/15, which respond differently. The sensitivity of cells to autophagy disruption was also independent of *TP53 s*tatus. For example, HCT116 cells have a wild type *TP53* while Caco-2/15 express mutated *TP53*. Others have reported that FOXO3a-mediated expression of the pro-apoptotic protein BBC3/PUMA increases sensitization of autophagy-deficient HCT116 cells to MDM2 inhibition^52^. However, our RNA-Seq data did not identify an increased expression of PUMA in HCT116 cells. This could be due to the stage at which the present samples were collected or the fact that the hypomorphic phenotypes followed in the CAM assays did not lead to FOXO3a accumulation. Finally, STAT3 regulation by autophagy was previously found to confer sensitivity to autophagy blockade in triple-negative breast cancer cells^64^. Again, the impact of autophagy inhibition on STAT3 in HCT116 and SW480 cells herein did not explain their responses. The only common feature potentially associated with the different groups of responders was their different capacity to regulate their autophagic flux. Interestingly, the ability to induce autophagy upon MEK1/2 inhibition was recently associated with sensitivity to autophagy inhibition^39^.

Our findings in HCT116 and Caco-2/15 cells demonstrate that autophagy blockade was effective at decreasing tumor growth, as shown previously^52^. In *KRAS*^*G12D*^ driven lung cancer mouse models, deletion of *Atg7* or *Atg5* reduces cell proliferation, increases cellular death and reduces tumor burden^13^. Some of these effects were related to the release of p53 suppression, although tumor growth was also affected by *Atg7* deletion in the absence of *TP53*, suggesting p53 independent mechanisms^13^. Lung cancer cells are highly dependent on glutamine for growth and it was hypothesized that protein degradation by autophagy was necessary to replenish TCA cycle intermediates^24^. Interestingly, in autophagy-impaired HCT116 tumors, translation and TCA cycle related genes were the most downregulated clusters. Furthermore, HCT116 cells have previously been shown to display lower Spare Respiratory Capacity (SRC) when compared to SW480^65^, results successfully replicated herein for Caco-2/15 and LoVo (not shown). SRC represents the extra capacity available in cells to produce energy in response to increased stress or work^66,67^, while low SRC is indicative of mitochondrial dysfunction. Therefore, differences in metabolic adaptations to the tumor microenvironment in the absence of autophagy could contribute to the opposite tumor growth characteristics of the various CRC cell lines.

Few studies have analyzed the molecular mechanisms driving cancer cell hyperproliferation under autophagy inhibition. In autophagy-impaired SW480, we observed a strong increase in AKT and RICTOR protein levels. To our knowledge, this is the first example of a feedback loop leading to increased AKT and mTOR activity upon autophagy inhibition. Both AKT and RICTOR transcripts were upregulated in autophagy-deficient cells. The *FOXO1* gene has been shown to induce RICTOR expression and to regulate AKT activity^68^. Similarly to FOXO3, FOXO1 may also be degraded by autophagy and hence autophagy inhibition could lead to increased FOXO1 levels and RICTOR expression in SW480. Other studies have shown that autophagy was required for ERK phosphorylation in NIH3T3 cells^69^ or for optimal receptor tyrosine kinase signaling in CRC cells^70^. Hence, modulation of these pathways may also potentially affect RICTOR expression. This is unlikely, however, given that no modulation of ERK or other MAPK was observed in the present phosphoarray analysis. However, GSK3-inhibiting phosphorylation was increased in the present phosphoarray and RICTOR can be targeted to proteasomal degradation by GSK3-dependent phosphorylation^71^. Hence, lower GSK3 activity could also promote increased RICTOR levels. Higher mTORC2 activity mediated by increased RICTOR expression may also lead to the observed AKT increase^72^. While no increase in mTORC1 components was observed in SW480 cells, two downstream targets of mTORC1 were nonetheless found to be hyperphosphorylated, suggesting higher mTORC1 activity. Interestingly, p62 regulates the mTORC1 complex and p62 overexpression is sufficient to increase mTORC1 activity^73^. p62 accumulation was observed upon autophagy inhibition in both HCT116 and SW480 cells. However, HCT116 cells have high basal mTORC1 activity^74^, while SW480 and LoVo, on the contrary, display low basal mTORC1 activity^74^. Hence, small differences in p62 levels could hypothetically translate into a significant effect on mTORC1 activity in these two cell lines. One drawback of our study is that it solely used SW480 cells to define the role of ATG5 and RAB21 in cancer cell hyperproliferation. Nonetheless, we believe that our findings provide a conceptual framework that could be tested in a broader scope of cell lines. While our study focused on colorectal cancer cells, the effects observed here might be applicable to other types of cancers, has demonstrated for the synergistic effects of MEK and autophagy inhibition in multiple types of cancer^39^.

From an autophagy and cancer standpoint, the present study conveys two important elements. First, colorectal cancer cell responses to autophagy inhibition cannot be predicted from their driver mutations, nor from their molecular subtypes^63^, but could potentially be based on their ability to upregulate autophagy upon stress. Second, cell hyperproliferation observed in autophagy-inhibited cells requires AKT and mTORC2 activity. Given that high expression levels of RICTOR are associated with poor prognosis in CRC^75^ as well as a variety of other cancers, our findings have clinical implications since therapeutic autophagy inhibition in cancer clinical trials is currently ongoing. Although the genetic determinants leading to such a response remain to be defined, our work highlights that including catalytic mTOR inhibitors in such trials could help generalize the impact of autophagy inhibition.

## Materials and Methods

### Cell culture

All cell lines used in this study were acquired from ATCC, apart from HIEC and Caco-2/15 which were from N. Rivard. CRC cells were grown in Dulbecco**’**s Modified Eagle Medium (DMEM)(Wisent, St-Bruno, Qc, Canada) or in 50% DMEM/50% Ham**’**s F12 media (Wisent) for LoVo and T84 cells. Both media were supplemented with 10% Fetal Bovine Serum (Wisent) and cells grown at 37 °C in 5% CO_2_. Plasmid transfection was performed using JetPRIME (Polyplus transfection, Illkirch, France), following the manufacturer**’**s instructions. siRNAs were transfected at a final concentration of 20 nM, except in HT29 (10 nM), using Dharmafect 1 and by following the manufacturer**’**s instructions. siRNAs were purchased from Dharmacon: RAB21(1) (J-009450-05), RAB21(2) (J-009450-08), ATG5 (J-004374-08) and VAMP8 (J-013503-05). Bafilomycin A1 was used at 200 nM in all experiments and cells were incubated either in normal growth media, in full starvation media (EBSS) or in glucose starvation media (DMEM without glucose, supplemented with 10% FBS) for the indicated time.

### Chorioallantoic membrane assays

Fertilized eggs from white leghorn chicken were obtained from Public Health Agency of Canada (Nepean, ON, Canada). Ethics approval was obtained from the Ethics Committee on Animal Research of the University of Sherbrooke and all experimental procedures involving embryos were conducted in accordance with the regulations of the Canadian Council on Animal Care. A total of 6 × 10^6^ CRC cells were plated in a 100 mm dish and transfected the following day with control, ATG5 or RAB21 siRNA (both RAB21 siRNA were used independently in CAM assays with similar results). Forty-eight hours after transfection, cells were resuspended at a concentration of 1 × 10^6^ cells per 10 µl and mixed with an equal volume of Matrigel (Corning, NY, USA). MK2206, Torin2, Rapamycin (Cayman Chemical, Ann Arbor, MI, USA) or DMSO was added in the cell/Matrigel mixture when stipulated in the figure. Twenty µl of the final mixture was grafted onto the chorioallantoic membranes of 7-day-old ex ovo chicken embryos. Drug treatment was repeated 72 hours after grafting by dilution in PBS and addition of a 20 µl drop on each tumor. Embryos were maintained at 37 °C for a total of 5 or 7 days after which tumors were removed using surgical forceps. Images of each tumor were acquired with a Leica M80 stereomicroscope coupled to a Leica EC3 digital color camera. Surface measurements were performed by averaging the volume (height*width*width) of each tumor using ImageJ.

### Immunohistochemistry

Tumors were fixed in 4% PFA immediately after excision from the CAM. Tumor processing (paraffin embedding, sectioning and hematoxylin/eosin staining) was performed by the Université de Sherbrooke histology core platform. Tissue sections were rehydrated and treated with 0.01 mol/L citrate, pH 6.0, and immunohistochemical staining was performed according to the standard avidin-biotin immunoperoxidase complex technique using anti-KI67 (1:200, GTX16667, Genetex, Irvine, CA, USA) or p62 (1:100, 7695, Cell Signaling Technology, Danvers, MA, USA) primary antibodies or isotype-matched negative controls and diaminobenzidene (Vector Laboratories Inc., Burlingame, CA, USA) as substrate. For quantitative immunohistochemistry, photomicrographs of the tumors were acquired using a Nanozoomer 2.0RS at 20X magnification (Hamamatsu, Shizuoka, Japan) and cells positive for KI67 or p62 staining in four representative areas for each tumor were counted or their intensity measured using CellProfiler.

### Cell viability

Cell viability was quantified using the CellTiter-Blue Assay (Promega, Madison, WI, USA) following the manufacturer’s instructions. Briefly, 1 × 10^5^ cells were plated in 96-well black plates. For glucose or oxaliplatin (10 µM) treatments, the drug was added, or starvation was initiated 24 hours following siRNA transfection and cell viability was assessed after 48 hours of treatment. Reagent was added (20 µl/well) and cells were incubated for 3 hours before recording of fluorescence at 560_ex_/590_em_ on a FlexStation 3 (Molecular Devices, San Jose, CA, USA) plate reader. Data was normalized to scramble controls for each cell lines (ratioed to 1) in Figure S3C and D. Hence, values lower then 1 indicate decreased cell proliferation in *ATG5*- or *RAB21*-depleted cells compared to the scramble control under starvation or oxaliplatin treatment.

### Colony formation assay

Cells were seeded in order to obtain isolated HCT116 – 500, SW480–1000, Caco-2/15, LoVo and T84–2000 cells in 24-well plates. Clones were imaged on a CloneSelect imager (Molecular Devices) 24 hours and 96 hours’ post-transfection. Colonies (formed by single cells) of more than 10 cells were counted. All experiments were performed in triplicate.

### Western blot analysis and Phosphoprotein array

For autophagy measurements, 5 × 10^5^ CRC cells were plated in 6-well plates. Cells were grown for 48 hours prior to treatments. Whole-cell extracts were prepared by lysis in RIPA buffer and proteins analyzed by Western blots. For assessment of protein levels in CAM tumors, tumors were removed and frozen in liquid nitrogen. Tumors were transferred to RIPA buffer and proteins extracted following 2-min homogenization with stainless steel beads at 50 oscillation/sec using a Tissuelyser LT (Qiagen, Hilden, Germany). Protein extracts were sonicated for 10 seconds. Protein concentration was measured with a BCA assay (Thermo Scientific Pierce, Waltham, MA, USA) prior to dilution in 5x Laemmli loading buffer. Equal amounts of proteins were resolved on SDS-PAGE gel and transferred onto PVDF membranes. Membranes were probed using the following antibodies: anti-tubulin DM1a (1:2500, T9026, Sigma-Aldrich, Darmstadt, Germany), anti-p62 (1:1000, sc-28359, Santa Cruz, Santa Cruz, CA, USA), anti-RAB21 (1:2000, R4405, Sigma-Aldrich), and from Cell Signaling: anti-PARP (1:2000, 9542), anti-Caspase3 (1:1000, 9662), anti-LC3B (1:4000, 3868), anti-p-mTOR (S2448) (1:1000, 5536), anti-mTOR (1:1000, 2983), anti-RICTOR (1:1000, 2114), anti-Raptor (1:1000, 2280), anti-p-S6K1 (T389) (1:1000, 9234), anti-p-4E-BP1 (T37/46) (1:1000, 2855), anti-p-AKT (T308) (1:1000, 13038), anti-p-AKT (S473) (1:1000, 9018), anti-p-AKT (T450) (1:1000, 9267), anti-pan-AKT (1:1000, 4691), anti-p-PRAS40 (1:1000, 2997), anti-P-NRDG1 (1:1000, 3217), anti-P-SGK1 (1:1000, 5599), anti-GAPDH:HRP (1:2000, 8884); followed by anti-mouse or anti-rabbit conjugated to horseradish peroxidase (1:20 000, Jackson ImmunoResearch, West Grove, PA, USA) and imaged on a Chemidoc MP (Bio-Rad, Hercules, CA, USA). All uncropped tif files exported from Image Lab for the various immunoblots presented in the manuscript are shown in supplemental Fig. 6. Western blot quantification was performed using the image lab software (Bio-Rad). Multiple independent experiments (Figs 1, S1–S3) or independent tumors (Figs 3, 5, 6 and S4) were quantified for all depicted graphs. Non-saturated bands were utilized for all quantifications. For image representation, all images depicted in the various figure panels were from non-saturated images. Contrasts were adjusted directly and linearly using ImageLab and images were exported in high resolution tiff (600 dpi). Crops were performed in Photoshop CC2019 and figures assembled in Illustrator CC 2019. Crops were done to ease figure presentation. Images from different gels were separated either with a line of were assembled in different blocks in the various figures and noted in the figure legend.

For the phosphoprotein array (R&D Systems, Minneapolis, MN, USA), 5-day tumors on CAM were pretreated with a protease inhibitor mixture (85 mM NaF, 35 mM β-glycerophosphate, 35 mM sodium pyrophosphate) for 30 minutes prior to excision. Protein extraction was performed as described above in the manufacturer’s lysis buffer. Protein array was carried out using 500 µg of proteins per condition, according to the manufacturer’s protocol.

To assess the role of RAB21 on CRC autophagy, 50 000 cells per well of a 24-well plate were seeded 24 hours before siRNA transfection. Experiments were performed 72 hours following siRNA transfection, and cell lysis was performed as detailed above.

### Immunofluorescence, confocal microscopy and image analysis

A total of 100 000 cells were plated on glass coverslips (#1.5) and grown for 24 hours. Bafilomycin A1 (0.2 µM) was added 4 hours prior to fixation. Starvation was performed in EBSS for 1 hour. Cells were fixed for 15 minutes on ice in 100% methanol. Cells were blocked and permeabilized for 60 minutes at room temperature in PBS containing 5% goat serum and 0.3% Triton X-100. Cells were then incubated in anti-LC3B (1:300, 3868, Cell Signaling) overnight at 4 °C in PBS containing 1% BSA, 0.3% Triton X-100. Secondary antibody incubation was performed at room temperature for 1 hour. Coverslips were mounted in DAPI containing mounting media (ThermoFisher) and subsequently imaged. All images were acquired on an LSM880 Zeiss confocal microscope using a 40×/1.4 NA plan Apo oil objective, with a 1.5 x zoom setting. Images were acquired with settings minimizing pixel saturation and analysis was performed on CellProfiler as in^44^. Endogenous LC3 punctae were identified using the identifyprimaryobject module. For the mCherry:GFP:Atg8 analysis, cells were fixed for 15 minutes in PFA 4%, coverslips were mounted in DAPI containing mounting media and red and red/green (orange) objects were counted manually.

### Transmission electron microscopy

Cells were pelleted and fixed in 1.5% glutaraldehyde in 0.1 M cacodylate buffer, pH 7.4 for 30 minutes followed by overnight fixation in 2.5% glutaraldehyde in the same buffer at 4 °C. Cells were post-fixed in 1% osmium tetroxide in 0.1 M cacodylate buffer for 1 hour and stained in 1% uranyl acetate for 16 hours at 4 °C. Cells were then dehydrated in ethanol and propylene oxide, embedded in epoxy resin, sectioned at 80 nm and mounted on copper grids. Grids were poststained with 2.7% lead nitrate/3.5% sodium citrate. Images were acquired on a transmission electron microscope (HITACHI H7500). Double membrane structures were counted as autophagosomes, while electron dense structures, with no apparent double membranes were counted as autophagic vesicles (amphisomes or autolysosomes).

### RNA-Seq

Three tumors were pooled per condition. Tumors were grinded into a fine powder in liquid nitrogen using a mortar and pestle. RNA was extracted using the RNeasy Mini Plus kit (Qiagen) following the manufacturer’s instructions with the optional on-column DNase digestion. Quality of the extracted RNA samples was assessed using the Agilent Bioanalyzer (Agilent, Santa Clara, CA, USA). All samples had a RNA Integrity Number (RIN) > 8.7. Library preparation and sequencing were performed at the McGill University and Génome Québec Innovation Centre using the NEB/KAPA stranded mRNA lib prep kit and sequenced on an Illumina HiSeq (Illumina, San Diego, CA, USA). Fastq files were verified for quality using FastQC (v0.11.4) and trimmed using Trimmomatic (v0.32) (with TRAILING:30) to remove adaptors and portions of low-quality reads. Read pairs were then aligned to the human genome build hg38 using an annotation file obtained from Ensembl (v 87) with the splice-aware RNA-seq aligner STAR (v2.5.1)^76^ using default parameters. Reads were also aligned separately on the Gallus gallus-5.0 genome assembly with the annotation file obtained from Ensembl (v 87) to verify the degree of contamination of the samples with egg tissue. Aligned reads were then assigned to genomic features using featureCounts from Subread (v1.5.2)^77^. FeatureCounts parameters were set to (-C,–largestOverlap, -p, -B, -s1,–minOverlap 10, -M). Count files produced by featureCounts were then compared between controls and knockdowns for SW480 and HCT116 cells separately for differential expression analysis using DESeq. 2 (v1.10.1)^78^. GSEA analyses were performed using the open source software (GSEA, v2.0, Broad Institute, MA, USA)^79^.

### Statistical analysis

The values are expressed as mean ± SEM. Nonparametric Kruskal-Wallis One-way ANOVA, one sample T test or Unpaired T Test were used to compare experimental groups, as indicated in the figure legends. Grouped data were evaluated by two-way analysis of variance (ANOVA). P values of less than 0.05 were considered to be statistically significant and displayed. All the statistical analyses were assessed using the GraphPad Prism software (V8) on the various graphs.

## Supplementary information

Supplemental figures

Supplementary Table 2

## Data Availability

The raw reads from the RNAseq dataset generated in the current study are available in the Gene Expression Omnibus (GEO) repository, under accession number GSE134095. All reagents generated during the course of this study will be made freely available upon request to the authors.
