# Supplementary material for: Colorectal cancer cells respond differentially to autophagy inhibition in vivo
Source: Sci Rep. 2019 Aug 5;9:11316. doi: 10.1038/s41598-019-47659-7 (PMC6683171; doi:10.1038/s41598-019-47659-7)

Supplementary Information:

Colorectal cancer cells respond differentially to autophagy inhibition *in vivo*.

Annie Lauzier[1,2], Josiann Normandeau-Guimond[1,2], Vanessa Vaillancourt-Lavigueur[1,2], Vincent Boivin[1,3], Martine Charbonneau[1,2], Nathalie Rivard[1,2], Michelle S Scott[1,3], Claire M. Dubois[1,2] and Steve Jean[1,2*]

*Corresponding author:

Email: steve.jean@usherbrooke.ca

Telephone: 819-821-8000 Ext: 70450

FAX: 819-820-6831

[1]Faculté de Médecine et des Sciences de la Santé

[2]Department of Anatomy and Cell Biology

[3]Department of Biochemistry

Université de Sherbrooke

3201, Rue Jean Mignaul

Sherbrooke, Québec, Canada, J1E 4K8

Supplementary Information:

Supplemental Table 1: Major genetic alterations in various CRC cell lines.

Supplemental Figure 1: CRC cells feature different autophagic capabilities. (A) Basal LC3-II levels in various CRC cells measured by anti-LC3 immunoblot analysis. (B) Per cell quantification of the average number of LC3 punctae from experiments depicted in Figure 1E ($n \geq 41$ cells from 3 independent experiments); mean ±SEM. (C) Representative images of TEM analysis of SW620 and HT-29 cells in full media or under glucose starvation. From data presented in Figure 2C. (D) Per cell quantification of the average number of autophagosome per cells from experiments depicted in Figure 2C and S1C (n= 12 cells from 3 independent experiments); mean ±SEM. Statistical analyses (B and D) were performed using Unpaired T Test.

Supplemental Figure 2: *RAB21* and *VAMP8* are differentially required for autophagic flux in various CRC cells. (A) *RAB21* and *VAMP8* are required for full autophagic flux to various degrees in the different CRC cells. Anti-LC3 immunoblot of *RAB21* and *VAMP8* siRNA-depleted CRC cells grown in full media with or without Bafilomycin A1. Associated quantification of three independent experiments; mean ±SEM. Gels – vs + BafA1 regions of each gels were separated to ease visualization and are from the same membranes. (B) Knockdown efficiencies of *ATG5* and *RAB21* depletion before CAM implantation in various CRC cell lines 48 hours after siRNA transfection. Images from different gels were separated by spaces.

Supplemental Figure 3: *ATG5* and *RAB21* depletion does not affect cell viability of CRC cells *in vitro*. Cell viability measured by a resazurin assay. (A) siRNA depletion of *ATG5* and *RAB21* does not affect cell viability of colorectal cancer cells grown in full media (n= 3 independent experiments); mean ±SEM. Viability was assessed 72 hours following siRNA transfection (B) Oxaliplatin (10µM) treatment or glucose starvation affect CRC cell proliferation to various degrees (n=3); mean ±SEM. Viability was assessed 48 hours after starvation induction or oxaliplatin addidtion. Oxaliplatin or glucose starvation in (C) *ATG5*- or (D) *RAB21*-depleted cells weakly sensitize cells. Results shown are the mean of 6 independent experiments; mean ±SEM. Viability was assessed 72 hours following siRNA transfection (48 hours after treatments, see methods section). All columns were normalized to control siRNA transfection (not shown). (E) Clonogenic assays in four CRC cell lines in full media or under serum starvation. Results shown are the mean of triplicates in 3 independent experiments; mean ±SEM. Statistical analysis was performed using (C and D) Unpaired T Test followed by Holm Sidak multiple comparisons test. Dotted red line in panels B, C, D represents a ratio of 1 in order to ease comparison of the various columns.

Supplemental Figure 4: Autophagy is perturbed in *ATG5*- and *RAB21*-depleted tumors in CAMs. (A) Images of SW620 and HT29 cells treated with scramble, *ATG5* or *RAB21* siRNAs implanted in CAMs and grown for 7 days post-implantation. (B and C) Quantification of tumor growth depicted in A (n= 2 independent experiments); mean ±SEM. P62 (SQSTM1) accumulation in *ATG5* and *RAB21* knockdown CRC cell-generated tumors. (D) Representative images of immunohistochemistry for the autophagic marker p62 (SQSTM1) in *ATG5*- and *RAB21*-depleted HCT116, Caco-2/15, SW480 and LoVo tumors extracted 5 days post-implantation. Slides were counterstained with hematoxylin/eosin. (E) Quantification of p62 IHC staining intensities from

four random fields in 2 to 5 tumors per condition; mean ± SEM. Mesenchymal cells being abundant in Caco-2/15 tumors, quantification did not reveal the increased p62 staining observed in *ATG5*- and *RAB21*-depleted cells, although increased staining intensity can be observed in cancer cells compared to control. (F) Immunoblot analysis of p62 and Tubulin from multiple independent SW480 tumors extracted five-days post implantation in CAMs. (G) Ratio of integrated densities of p62 to Tubulin for at least 13 tumors per condition; mean ±SEM. Statistical significance for E) and G) was determined by One-way ANOVA.

Supplemental Figure 5: Histological assessments of CAM generated tumors and phosphoarray results on various kinases. (A) Hematoxylin-eosin staining of tumor sections isolated from CAM injected with HCT116, Caco-2/15, SW480 and LoVo cells transfected with control, *ATG5* or *RAB21* siRNAs. (B) Phosphoarray normalized ratios from three independent *ATG5*- and *RAB21*-tumors normalized to pooled scrambled control tumors. Each bar represents duplicates for each tumor.

Supplemental Figure 6: Uncropped .tif files exported from Image lab used for data representation throughout the manuscript.

Cell Line	MSI status	KRAS	BRAF	PIK3CA	PTEN	TP53
**SW480**	MSS	G12V	wt	wt	wt	R273H;P309S
**LoVo**	MSI	G13D;A14V	wt	wt	wt	wt
**SW620**	MSS	G12V	wt	wt	wt	R273H;P309S
**HT-29**	MSS	wt	V600E	P449T	wt	R273H
**HCT116**	MSI	G13D	wt	H1047R	wt	wt
**Caco-2**	MSS	wt	wt	wt	wt	E204X
**T84**	MSS	G13D;G38A	wt	G1624A	wt	G811T

Supplemental Figure 1

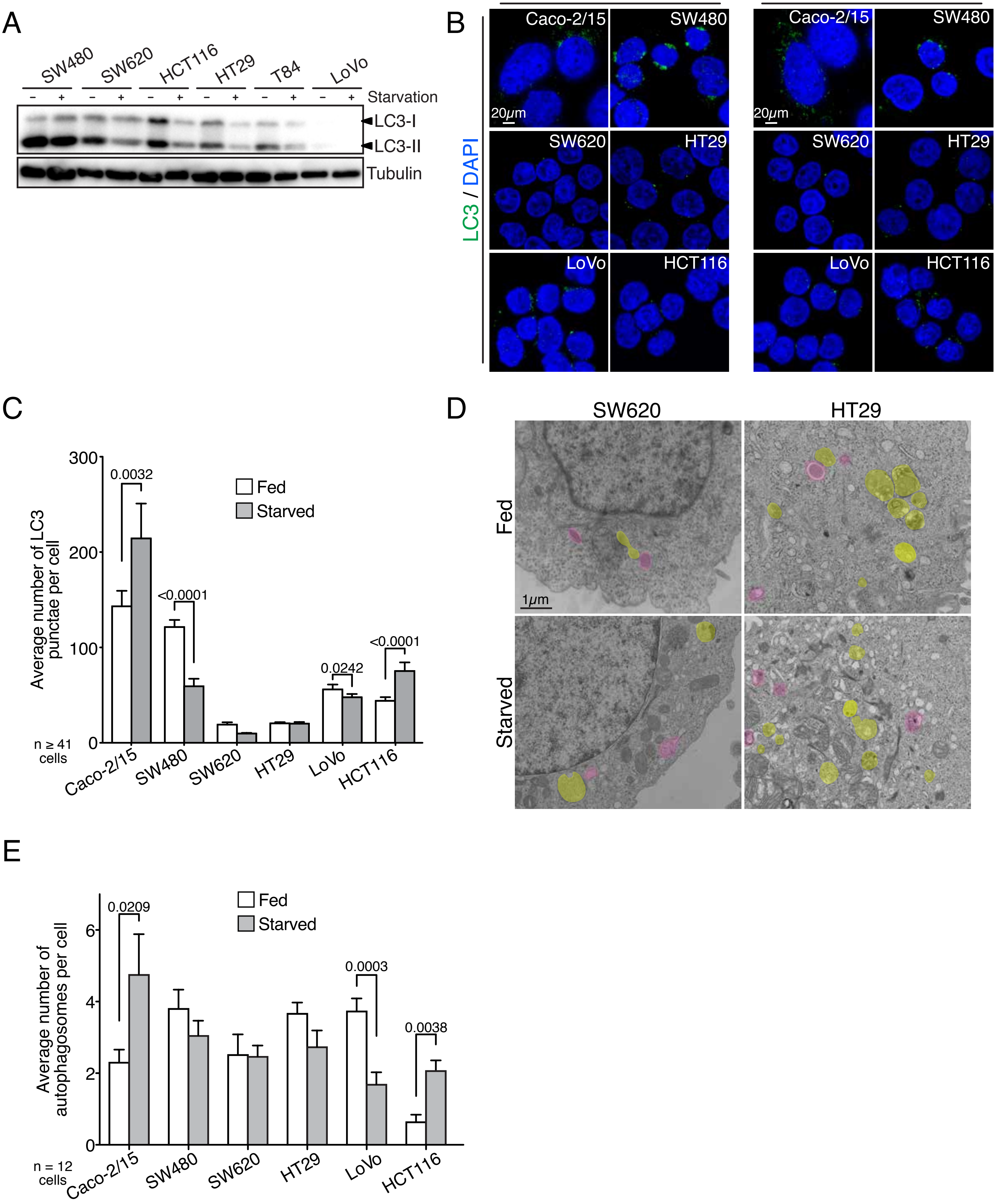

Supplemental Figure 2

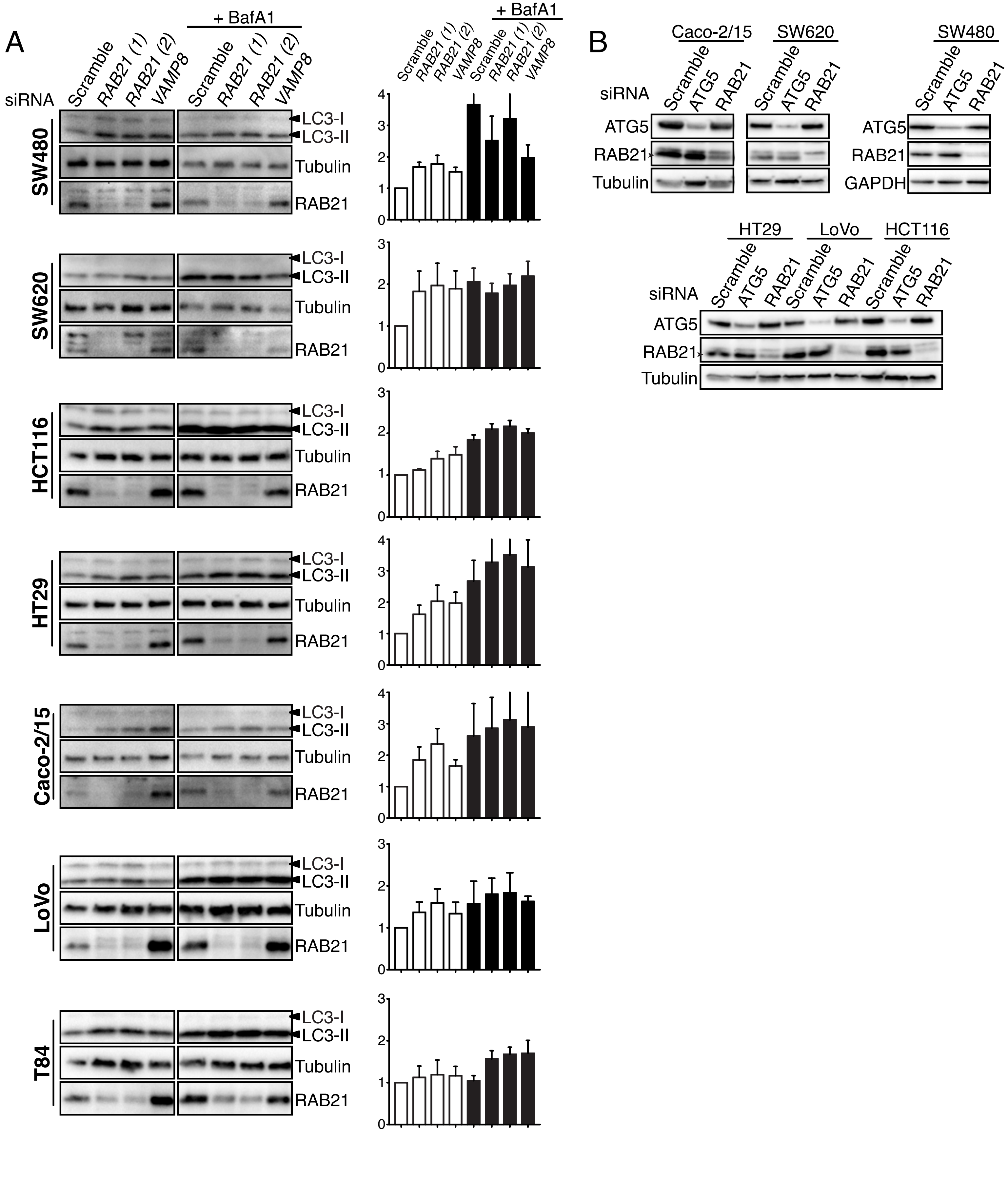

Supplemental Figure 3

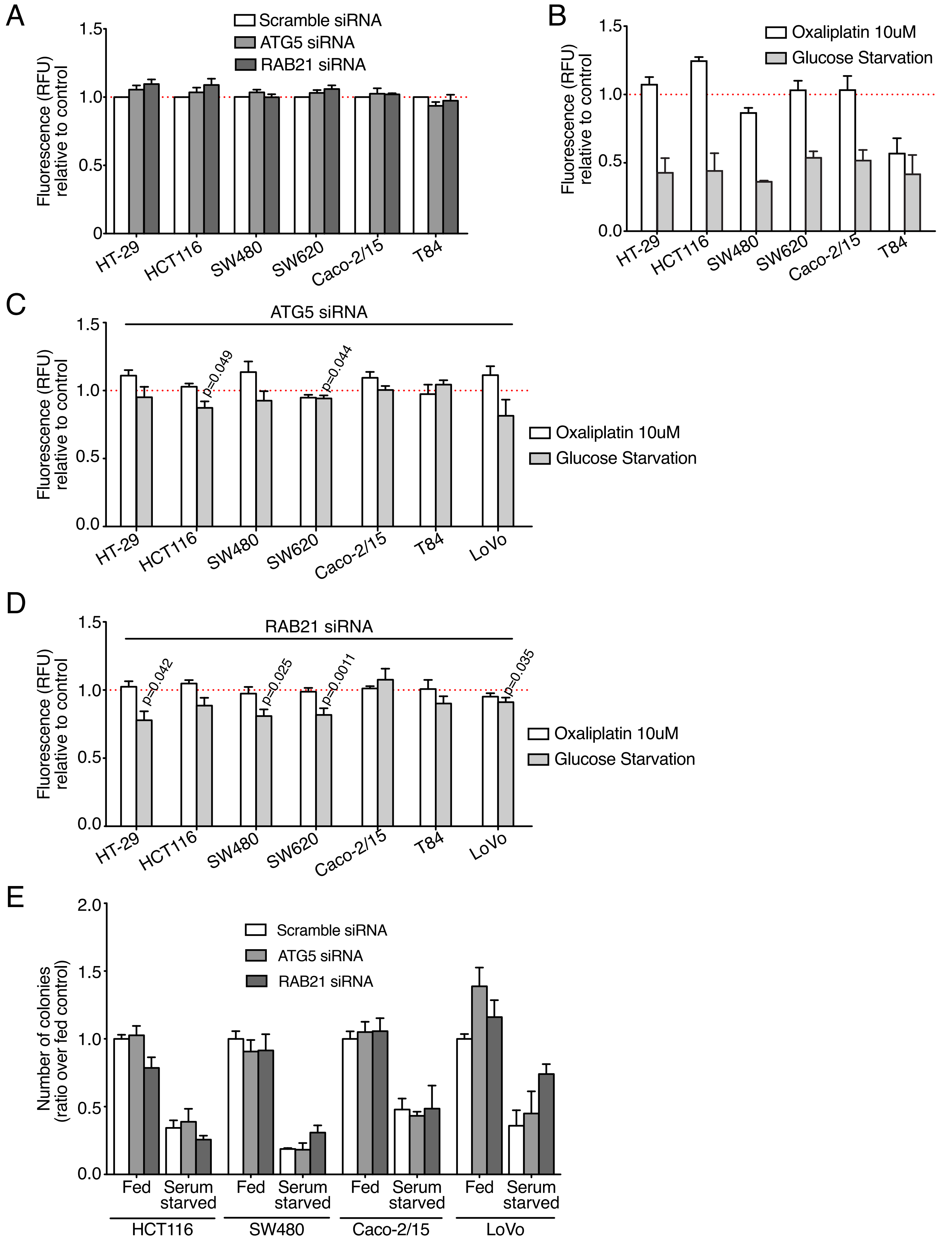

Supplemental Figure 4

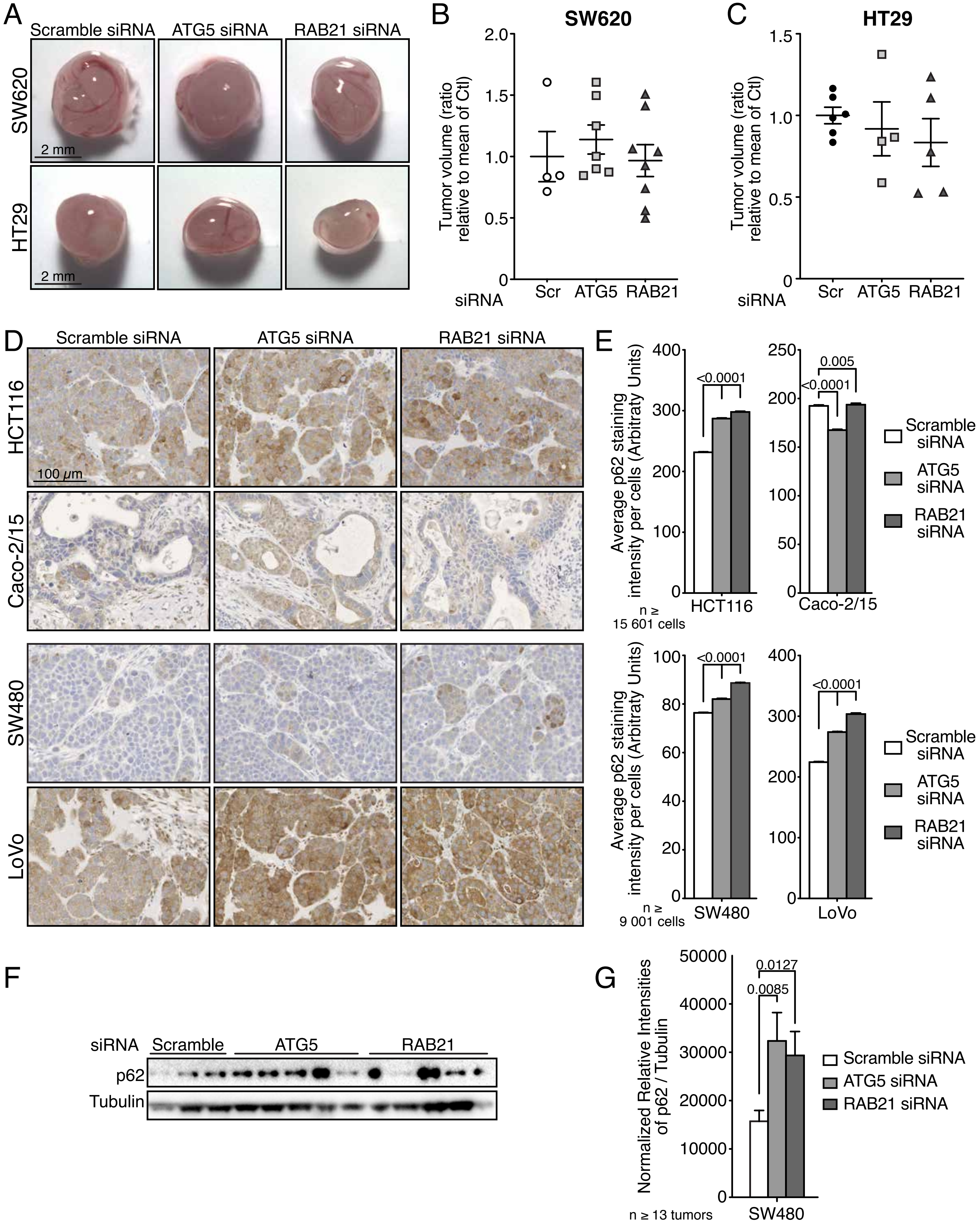

Supplemental Figure 5

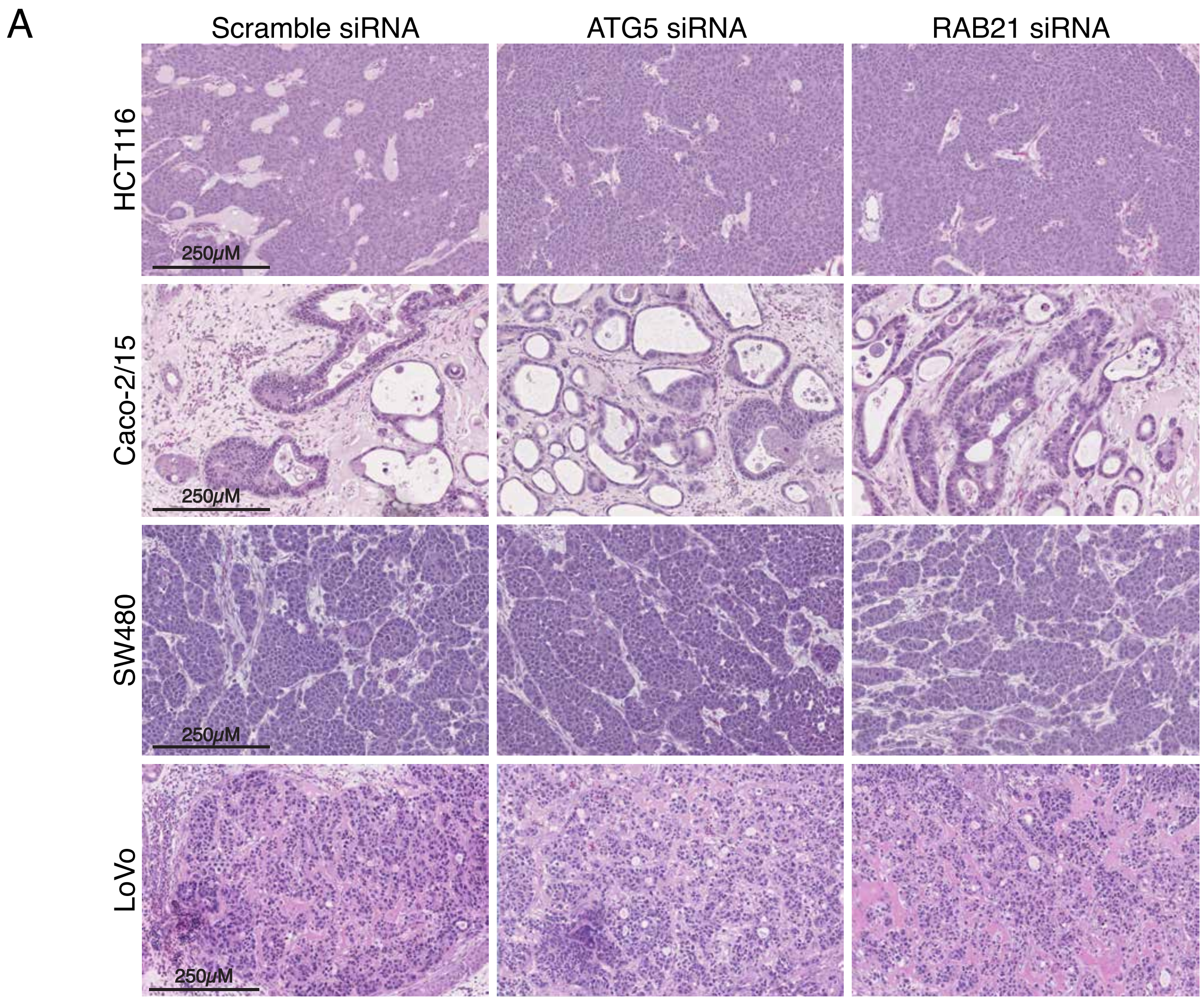

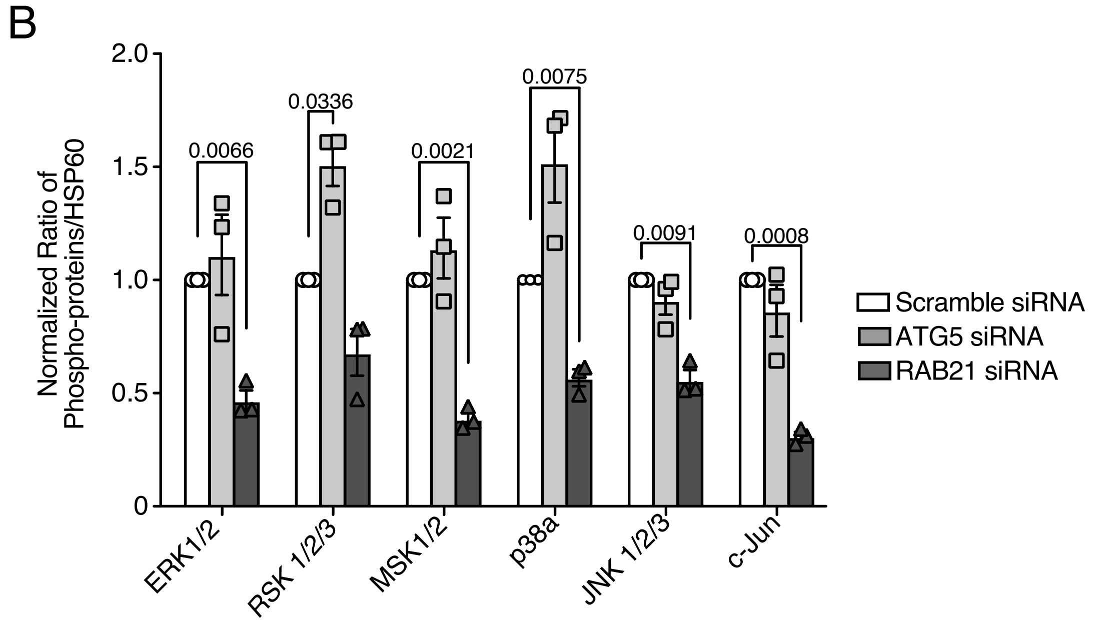

Supplemental Figure 6

Full length original western blots

Figure 1

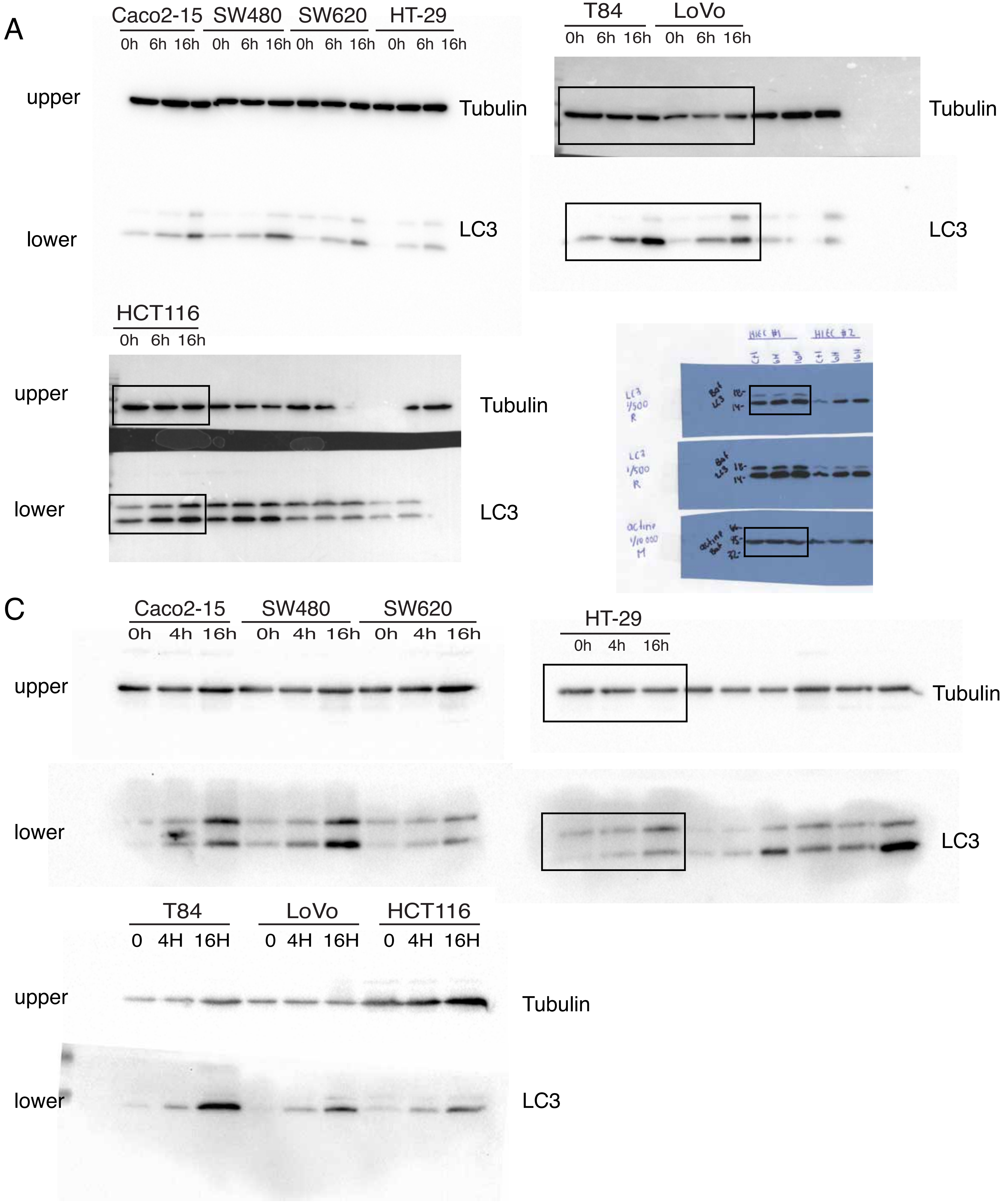

Supplemental Figure 6

Full length original western blots
Figure 3

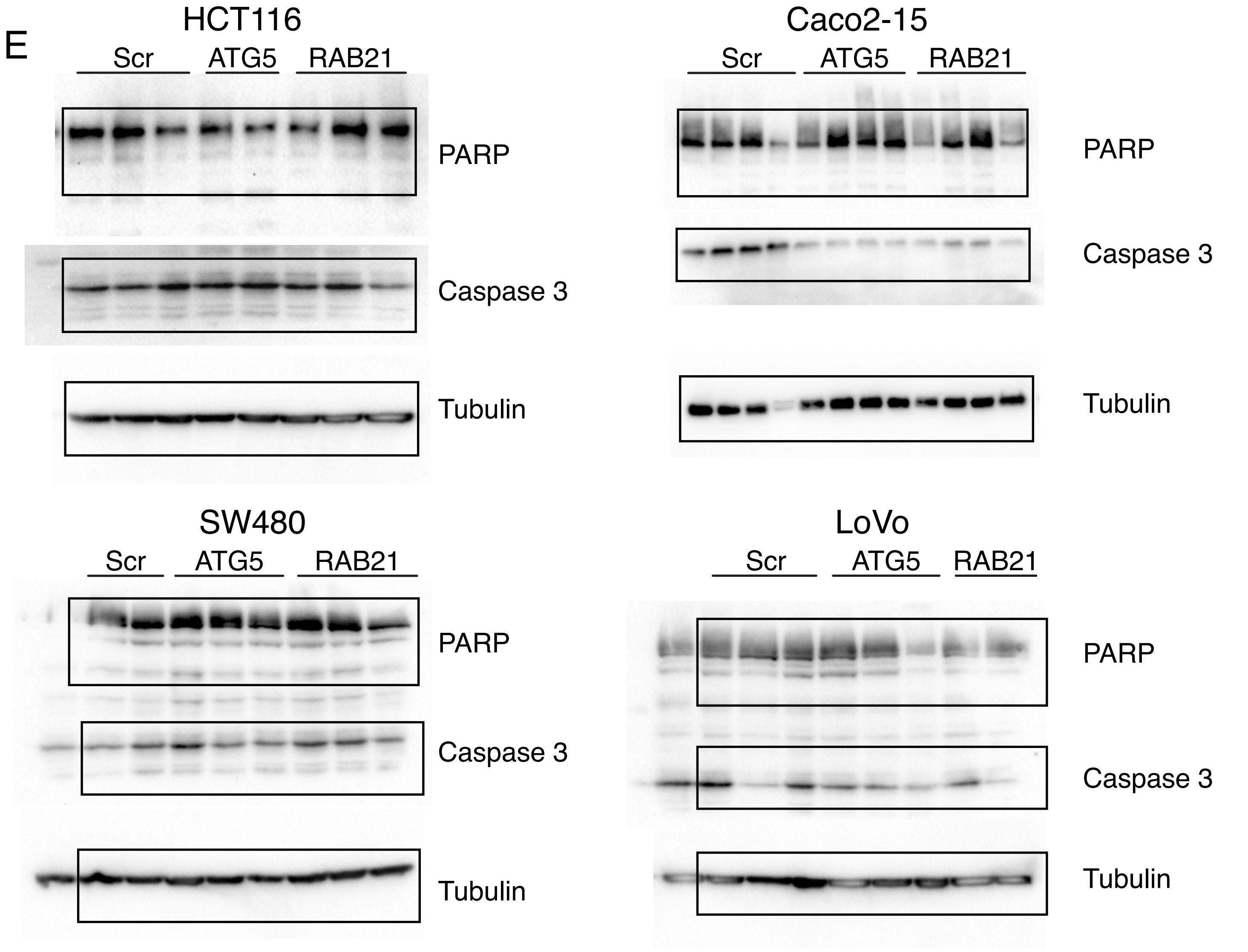

Supplemental Figure 6

Full length original western blots
Figure 5

B

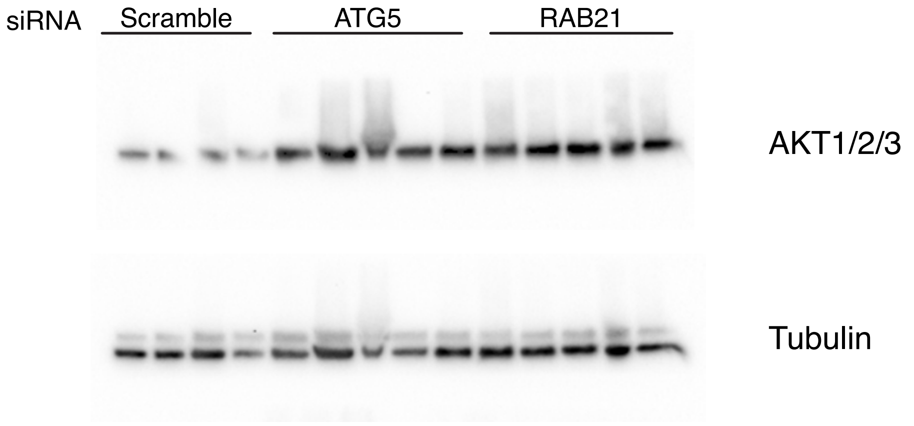

D

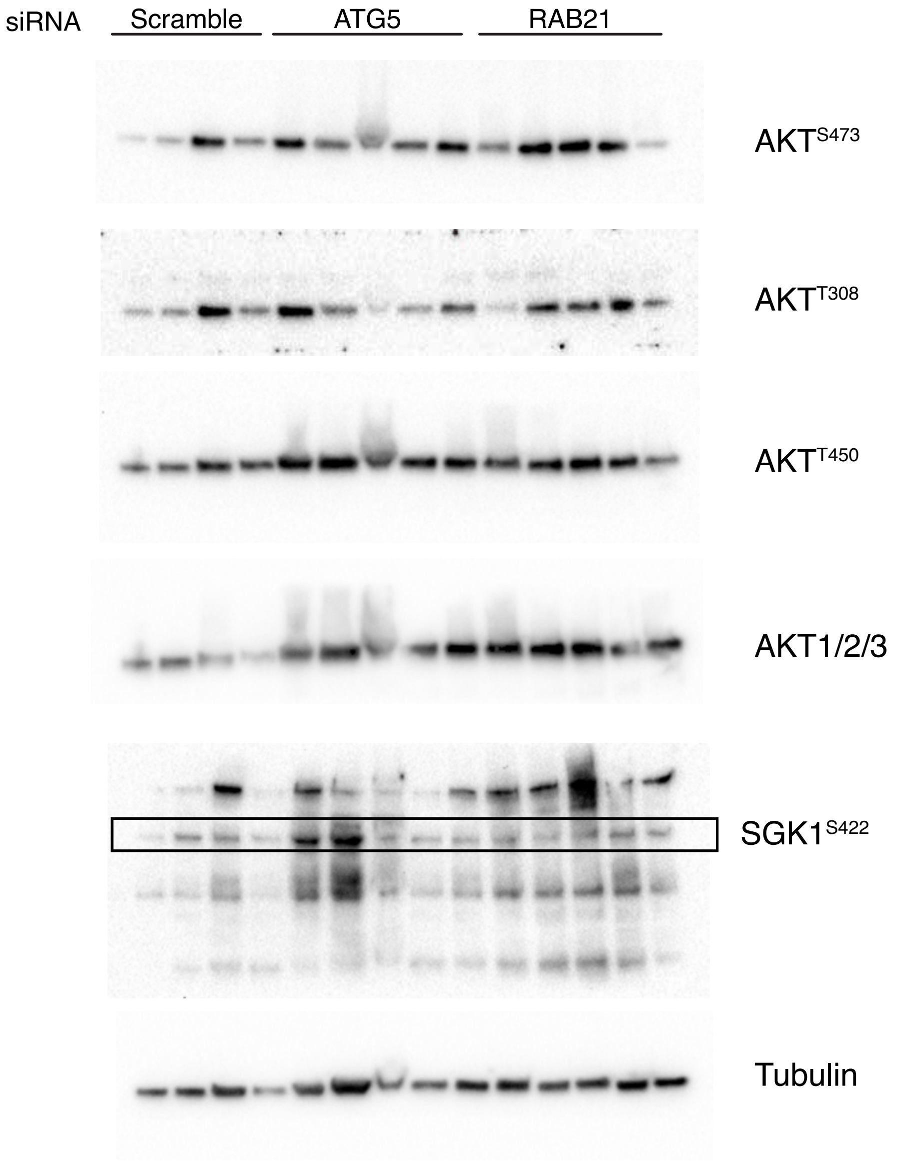

Supplemental Figure 6

Full length original western blots

Figure 6

A

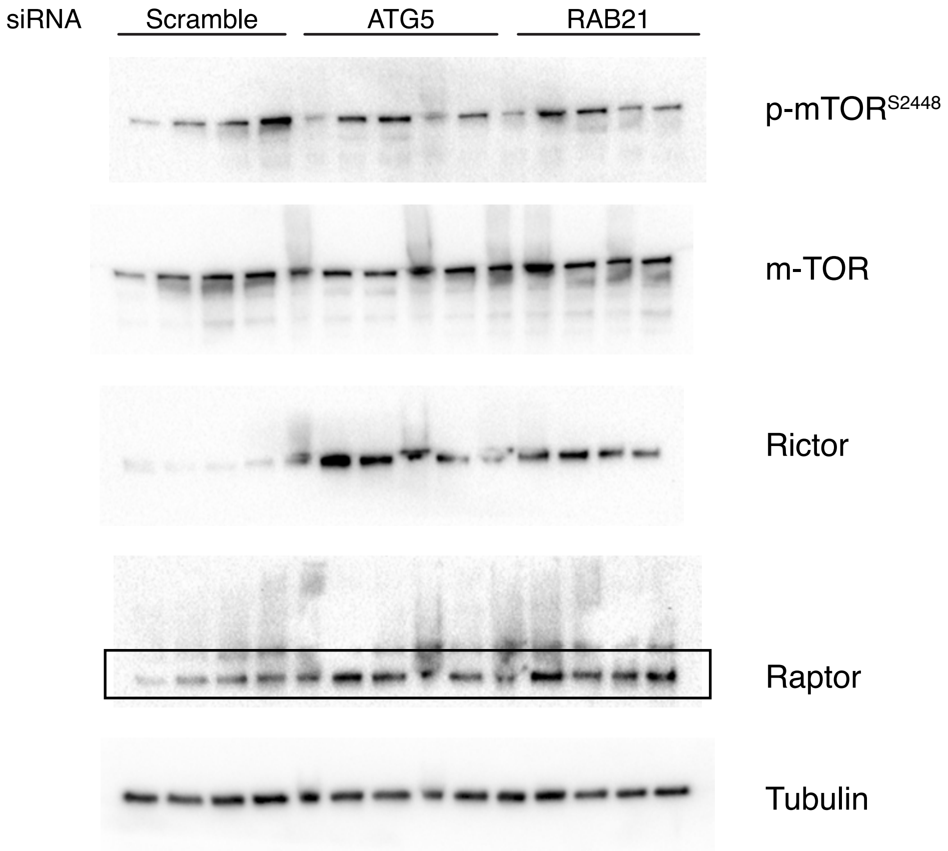

C

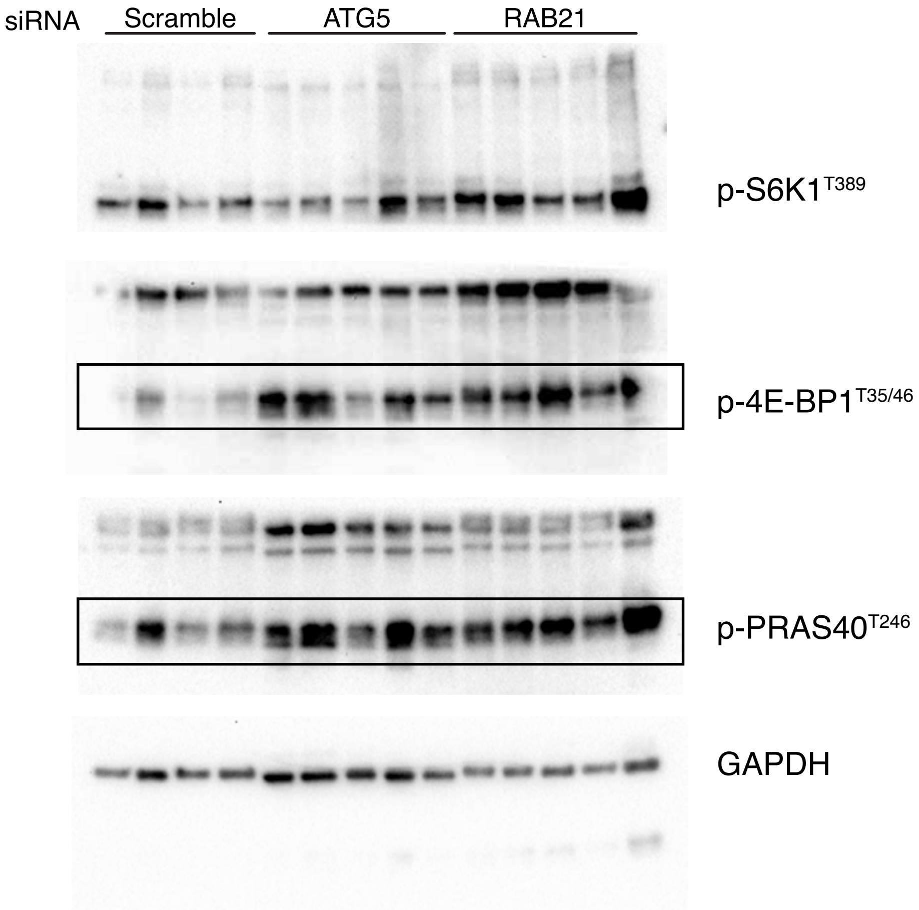

Supplemental Figure 6

Full length original western blots

Figure 6

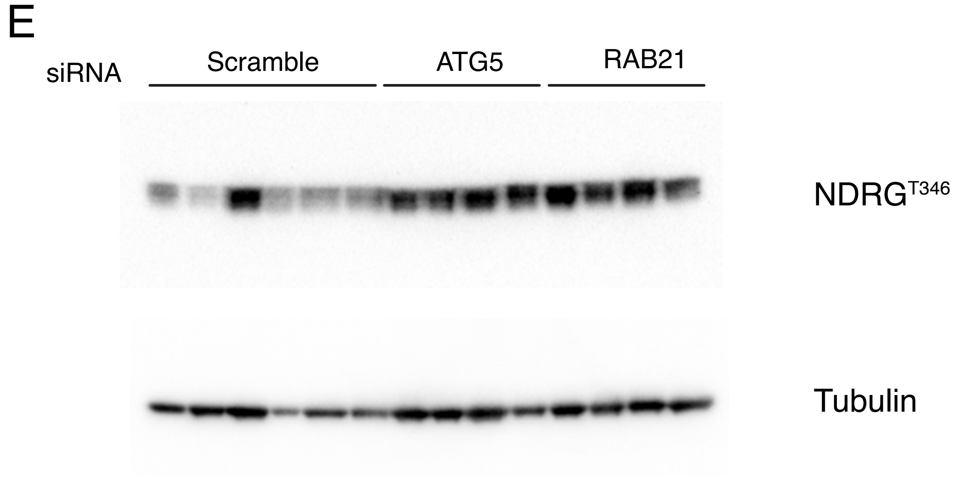

Figure 7

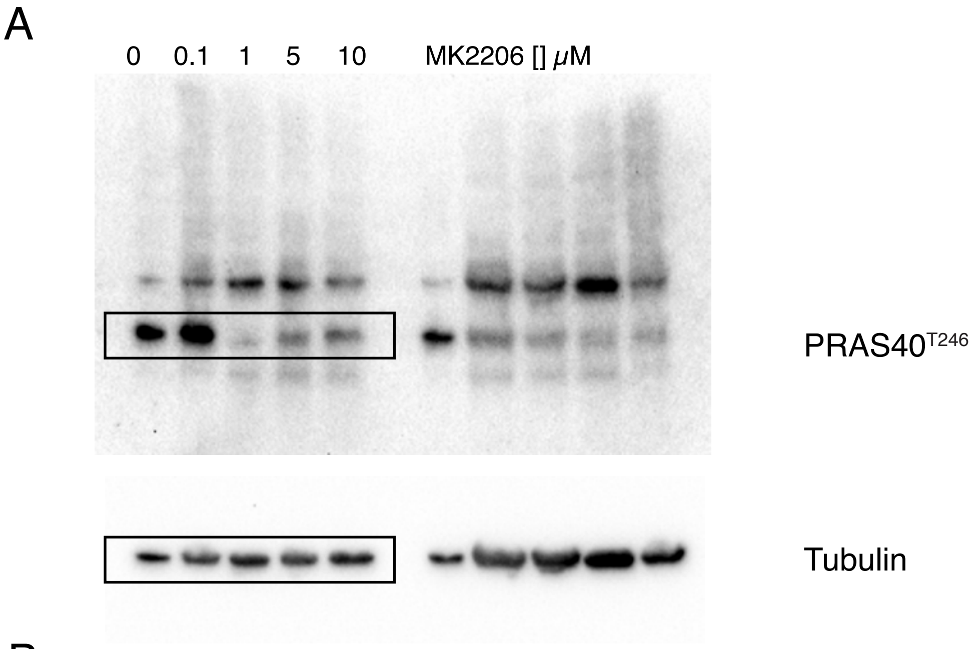

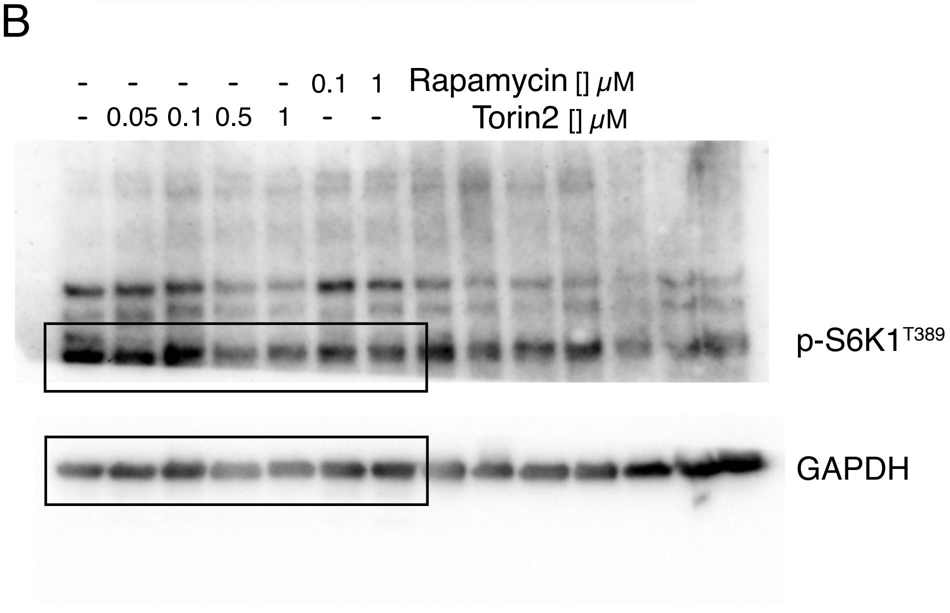

Supplemental Figure 6

Full length original western blots

Figure S1

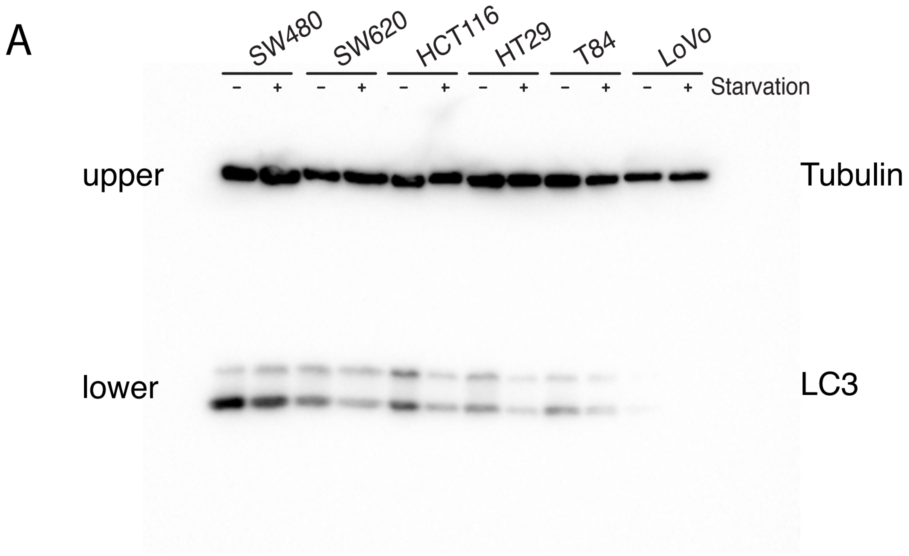

Figure S2B

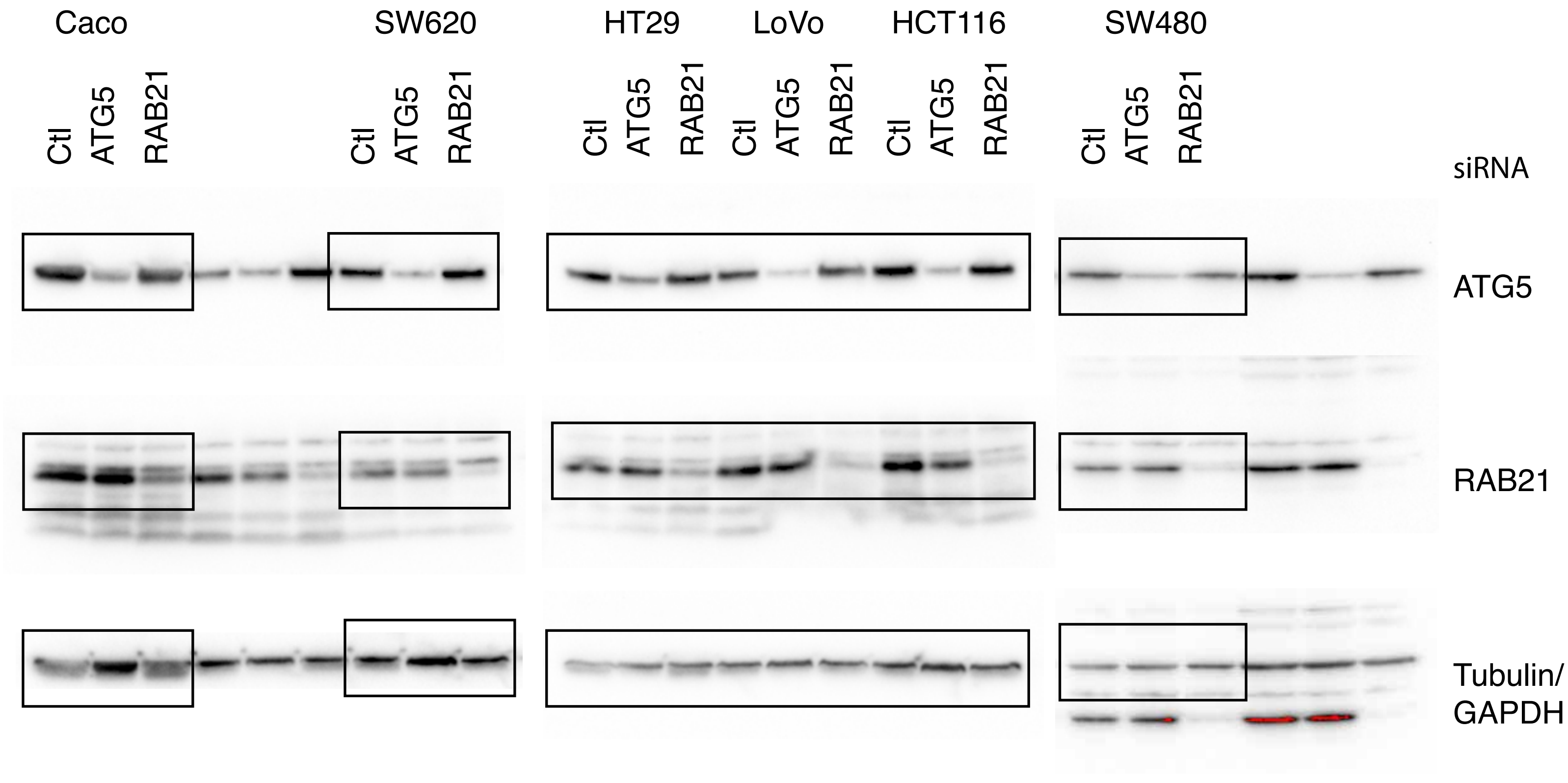

Supplemental Figure 6

Full length original western blots
Figure S2

A

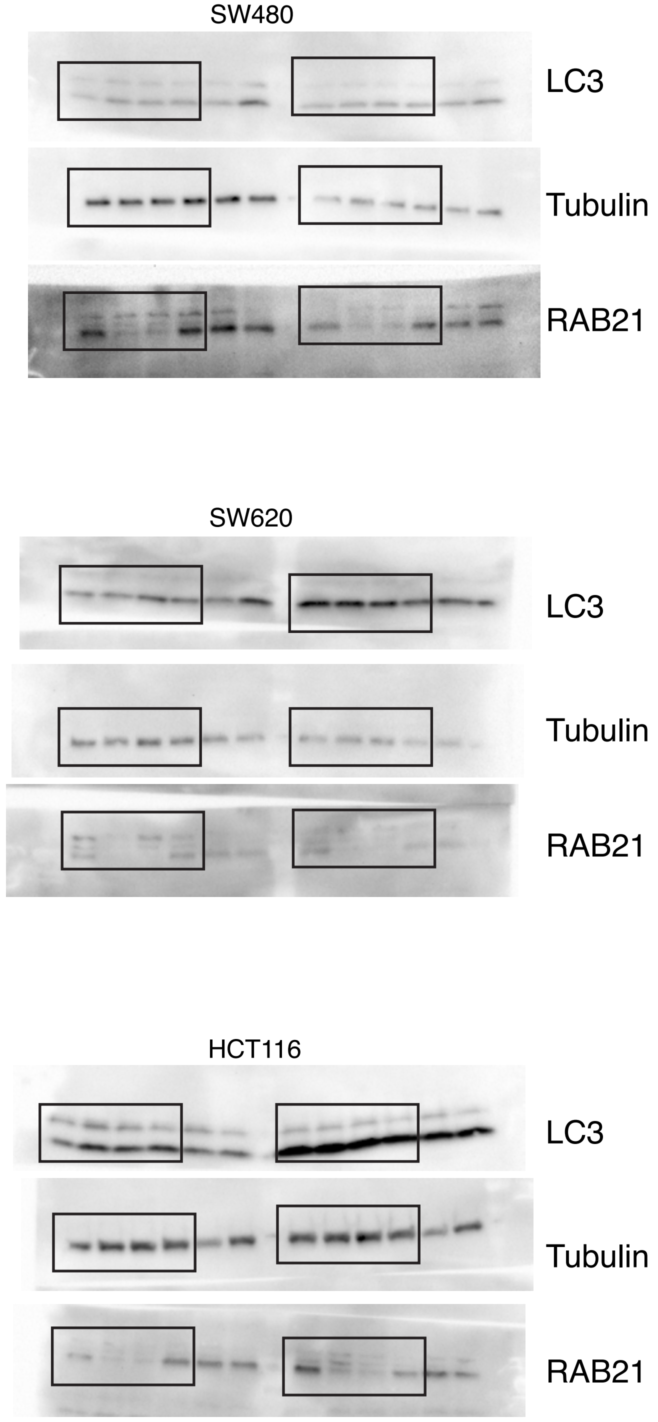

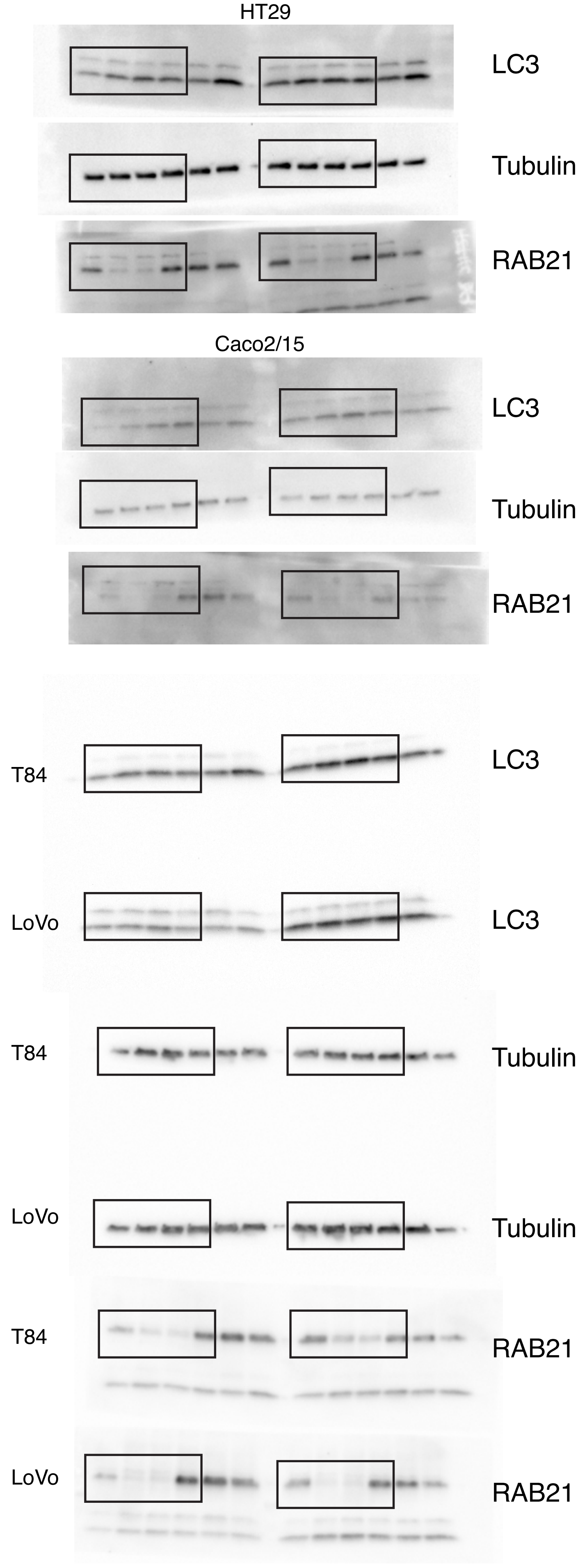

Supplemental Figure 6

Full length original western blots

Figure S4

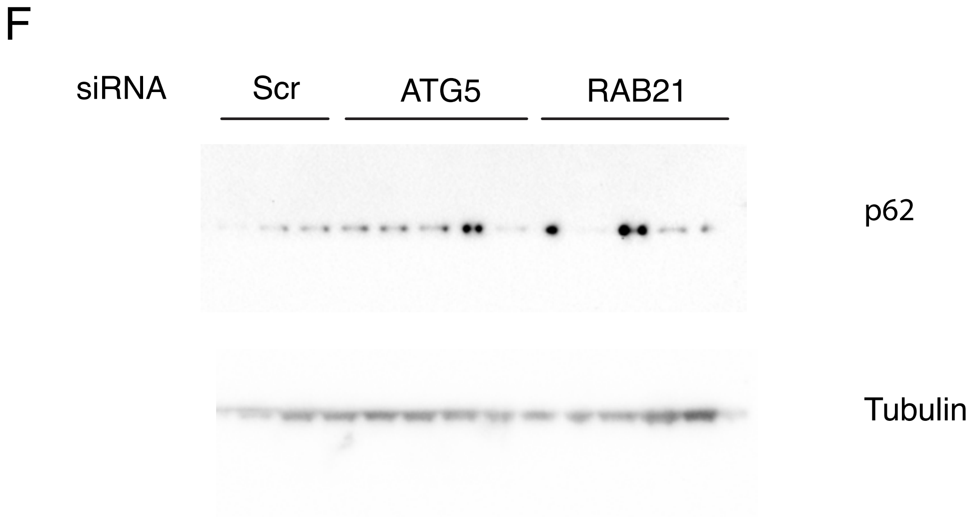

Supplement: Supplementary file 1 — Supplemental figures [file 41598_2019_47659_MOESM1_ESM.pdf]
